# Supplementary material for: Polarization Conforms Performance Variability in Amorphous Electrodeposited Iridium Oxide pH Sensors: A Thorough Surface Chemistry Investigation
Source: Sensors (Basel). 2024 Feb 1;24(3):962. doi: 10.3390/s24030962 (PMC10856937; doi:10.3390/s24030962)
Supplement: Supplementary file 1 [file sensors-24-00962-s001.zip › sensors-2814491-supplementary.pdf]

sensors

Supplementary Materials for

Polarization Conforms Performance Variability in Amorphous Electrodeposited Iridium Oxide pH Sensors: A Thorough Surface Chemistry Investigation

Paul Marsh [1,†], Mao-Hsiang Huang [1,†], Xing Xia [1], Ich Tran [2], Plamen Atanassov [3,4] and Hung Cao [1,5,6,*]

1 Department of Electrical Engineering and Computer Science, University of California Irvine, Irvine, CA 92697, USA; marshp@uci.edu (P.M.); maohsiah@uci.edu (M.-H.H.)
2 Irvine Materials Research Institute, University of California Irvine, Irvine, CA 92697, USA; ictran@uci.edu
3 Department of Chemical and Biomolecular Engineering, University of California Irvine, Irvine, CA 92697, USA; plamen.atanassov@uci.edu
4 Department of Materials Science and Engineering, University of California Irvine, Irvine, CA 92697, USA
5 Department of Biomedical Engineering, University of California Irvine, Irvine, CA 92697, USA
6 Department of Computer Science, University of California Irvine, Irvine, CA 92697, USA
* Correspondence: hungcao@uci.edu
† These authors contributed equally to this work.

Experimental Details

Substrate Fabrication

All IrOx films electrodeposited in this work were built atop substrates consisting of patterned metallic layers on SiO_2/Si wafers. 100 mm dia. Si wafers were purchased which featured orientation (100), were 500 µm thick, single-side polished, and featured 500 nm of thermally-grown SiO_2 (University Wafer, Inc., Boston, MA). These were given a basic solvent cleaning (acetone, IPA, N_2 drying) prior to use.

Metallization and patterning followed a typical liftoff lithography process. NR9-1500PY (Futurrex, Inc., Franklin, NJ) was spun onto the wafers at 3000 RPM for 40 s, with a ramp rate of 420 RPM/sec (WS-400B-6NPP, Laurell Technologies, North Wales, PA). The wafers were then soft baked by hot plate contact at 75 deg C for 2 min, exposed to 355 nm light at 10 mW/cm^2 for 28 sec, through a printed mask transparency (CAD/Art Services, Inc., Bandon, OR), in an optical mask aligner (Karl Suss MA6, SUSS MicroTec SE, Germany), and post baked by hot plate contact at 75 deg C for 5 min. After allowing the wafer to cool, NR9 was developed in RD6 (Futurrex) for ~5 s, with mild agitation. Once the liftoff layer was patterned, metallization was performed by either sputtering (DV-502M, Denton Vacuum, Moorestown, NJ) or electron beam evaporation (Temescal CV-8, Ferrotec, Santa Clara, CA). In the case of sputtering, 20 nm of Cr was deposited via 50 W of DC power (~1.0 Å/sec), followed by 200 nm of Au deposition via 20 W of RF power (~0.7 Å/sec). In e-beam evaporation, 20 nm of Cr was deposited via 20 mA at 7.3 kV, followed by 200 nm of Au deposition via 60 mA at 7.3 kV. Following metal deposition, NR9 was removed via sonication at 40 kHz in acetone for 5 min and in IPA for 5 min; afterwards, wafers were rinsed in DI water and dried by N_2. Several more layers of photoresist were added: 1 µm of Parylene-C was deposited by vapor deposition (PDS 2010, SCS, Indianapolis, IN) for use as a barrier layer and to prevent contact shorts and control pad surface area, while S1827 (Kayaku) was used as an etching pattern for Parylene-C and then re-deposited as a sacrificial layer during wafer dicing. Parylene-C was deposited at a starting pressure of less than 10 mTorr. S1800 deposition was as follows: spinning on at 3000 RPM for 30 sec with a 460 RPM/sec ramp; soft-bake by hotplate contact at 70 deg C for 3 min; un-patterned exposure for 30 sec; and developing in MF-319 (Kayaku) for ~25 sec with mild agitation. Some Parylene-C blistering beneath exposed S1827 took place but rarely resulted in liftoff

during etching. Etching of Parylene-C was conducted by O_2 reactive ion etching (Trion Technology, Tempe, AZ); briefly, 50 sccm O_2 flow was used at 300W for 120 s continuously, resulting in an etch rate near to 0.5 µm/minute. Wafers were diced into individual probes (7910 Uno, ADT, Horsham, PA) with a diamond resin-bond blade, 53 µm grit, ~200 µm thick. Any residual S1800 was then removed by solvent and DI rinses. Performing RIE until Parylene-C and S1827 was completely removed above Au was crucial, as electrodeposition would take place partially, or not at all, if polymer contamination was present. Final sample pads were 1 mm x 1 mm square; overall probe dimensions are provided in Figure S3.

Electrodeposition Process and Discussion

Electrodeposition was performed by cyclic voltammetry, with probe test pads immersed in an iridium-oxalate solution. Cyclic deposition has been shown to produce highly uniform, dense, and well-adhering IrOx films, and oxalate-based solutions are simple to produce and amenable to electrodeposition and long term storage [1]; pulsed deposition was initially attempted but proved difficult to tune properly and resulted in flaky, coarse films on smaller geometry, which extended beyond the bounds of the metallic substrate. While cyclic voltammetry is less time efficient, the slower polarization transitions produced more morphologically homogenous films and they were confined more locally to the metallic substrate. Iridium oxalate solutions were prepared based on Yamanaka's original recipe [2]. Briefly, 70 mg of anhydrous $IrCl_4$ (ArtCraft Chemicals, Altamont, NY) was stirred into 50 mL of DI water for 30 min; next, 0.5 mL of 30% H_2O_2 (H325, Fisher Chemical, Vernon Hills, IL) was stirred in for 10 min; after, 250 mg of oxalic acid dihydrate (247537, Sigma Aldrich, USA) was stirred in for an additional 10 min; finally, pH was set to 10.5 using K_2CO_3 (P1472, Sigma Aldrich) and the solution was allowed to rest for 4 days before being stored in a refrigerator for later use. Refrigerated shelf-life for the final solution was several months, if usage was kept to a minimum.

For deposition, Au/SiO_2 probes were used as working electrodes (WE), paired with Ag/AgCl glassy reference electrodes (RE) filled with 1 M NaCl, and 0.5 mm dia. Pt wire counter electrodes (CE) immersed in solution roughly 15 mm. WE contact pads were connected via Kelvin clip (3313, Adafruit Industries, New York, NY), RE and CE pins by alligator clip. Cyclic deposition was performed by commercial potentiostat (CHI 760E, CH Instruments, Austin, TX), using the following parameters: sweeping between -0.5 V and +0.7 V (vs Ag/AgCl in 1 M NaCl), for 2000 segments (1000 cycles), at 5 V/s scan rate, yielding a runtime per coating of ~8 min; initial scan polarity was driven from 0 V towards anodic (positive) potentials, final scan polarity was towards the cathodic (negative) limit. Prior to each use, the CHI was allowed to idle for 30 min, an internal hardware check was performed, and cable functionality was confirmed by voltage sweep across a 50 Ω resistor in a 3-wire configuration. All electrodes were fixed in repeatable cell spacing by printed ABS bracket, fitted to 50 mL low griffin beakers. Deposition temperature was controlled by Peltier cooler to 60 deg F.

All glassware for solution mixing and testing was cleaned by immersion in a 1:1 mixture of 10% HNO_3 and 15% H_2O_2 at 85 deg C for 12+ hours, followed by three boiling DI water baths, then N_2 drying, and stored upright with Parafilm covering (P7793, Sigma Aldrich) or upside down.

Calibration Testing

Research grade commercial buffers (Fisher Scientific) used for testing were as follows: the pH 4 solution was comprised of 0.05 M potassium hydrogen phthalate, formaldehyde, and methyl alcohol; the pH 7 solution was comprised of 0.05 M potassium phosphate monobasic and sodium hydroxide; the pH 10 buffer was comprised of 0.05 M disodium ethylenediaminetetraacetate dihydrate, potassium carbonate, potassium hydroxide, and potassium borate. Probes were immersed in solution along with Ag/AgCl (in 1 M NaCl) reference electrodes, spacing held constant by bracket, and immersed in DI water between each bath.

Potentiostatic Conditioning

For polarization of the IrOx film, a simple ionic solution of 10x phosphate buffered saline (PBS) was chosen. Chosen potentials were held for 180 s vs the same counter and reference electrodes listed in the electrodeposition section. Pt CE's were cleaned by acetone and IPA between runs. As with electrodeposition, potentiostat circuit and lead function tests were performed before each experimental day.

Surface Technique Parameters

XPS was used to analyze surface oxidation and infer surface species; all scans were run on an AXIS Supra (Kratos Analytical Ltd., UK), using monochromatic Al Kα excitation (1486.6 eV) through a 110 μm aperture (to avoid scanning the surrounding substrate). Ir 4f/5p spectra were obtained in the 43 to 72 eV range; O 1s spectra were obtained in the 526 to 540 eV range; Na 1s spectra were taken between 1065 eV and 1078 eV; C 1s between 280 eV and 325 eV; survey spectra were taken up to ~1490 eV. Survey spectra were collected using a step size of 1 eV and pass energy of 160 eV, core-level spectra were collected using a step size of 0.1 eV and pass energy of 20 eV. To avoid significant spectral alteration via ligand reduction, O scans were run prior to Ir scans. In general, scans were taken in the following order: survey scans, details scans, and valence scans. Scan resolution for wide scans, detail scans, and valence spectra were 160 eV, 20 eV, and 10-40 eV, respectively. The system's binding energy scale was calibrated to Au/Ag Fermi edge and core levels following Ar+ ion etching; the transmission function is shown in Figure S1. Because electrodeposited IrOx can present as semiconducting or metallic [3,4], the IrOx Fermi edge could not be trusted for use in charge referencing the resulting data via the work function method [5]. Instead, data is presented as-measured. In specific instances, it has been charge referenced to adventitious carbon, or AdC (aligning the main C 1s peak to an arbitrary location of 284.8 eV). Although this is known to be highly variable [6], it is only used to illustrate certain distribution patterns, and attention is called to each figure where the technique was used. Sample conductivity was sufficient to avoid the use of a flood gun. Fitting and analysis took place in the CasaXPS software package (http://www.casaxps.com/), and internal relative sensitivity factors for Kratos machines were used. Satellite estimation (discussed below) was done using the Fityk software package (https://fityk.nieto.pl/) [7].

Valence spectra was also obtained using He II excitation (40.8 eV); Fermi-edge and work-function measurements were taken by He I excitation (21.2 eV); low intensity XPS (LIXPS) was conducted near 1482 eV; in special cases, angle-resolved XPS was performed at fixed angles of 0°, 40°, 55°, and 70° and/or Ar^+ sputtering was performed in cluster mode for 5-10 s per pass. LIXPS proved too variable to be included, and He I cutoff provided limited information (see main text and Figure S15). Fitting procedures are discussed in a separate section, analysis procedures are discussed in the main text.

SEM images were taken at 150 - 330 kx magnification, 5 – 25 kV, secondary electron imaging; imaging was performed on a GAIA3 SEM-FIB (Tescan Orsay Holdings, a.s., Czech Republic). Prior to imaging, specimens were coated in 3-5 nm carbon (EM ACE600, Leica Microsystems, Germany). For EDS, both maps and point scans were conducted, generally at 20 kV with process times of 4 – 6. Scans were performed via a 150 mm^2 silicon drift detector (X-Max 150, Oxford Instruments, UK) and analyzed via the AZtec software package (Oxford). SEM and EDS were conducted within the same vacuum cell within the same instrument session.

As discussed in the main text and demonstrated in Figure S2, the IrOx films were thinner than that of the Au substrate; since both the IrOx and Au layers were ≤ 200 nm, XRD was taken in grazing-incidence parallel beam mode (GI-XRD), with a critical angle of 0.5 deg. Scans were done over a 2θ range of 20 – 80 deg in 0.05 deg steps at 0.1 deg/min, with samples rotated off the scan axis by 15-30 deg. They were conducted on a SmartLab diffractometer (Rigaku Corp., Japan).

Contact profilometry was used to measure film thickness, by comparing to local substrate thickness. Scan speeds were generally run at 0.15 μm/point in 6.5 μm vertical windows, using a 12.5 μm probe tip. All scans were run on a DektakXT profilometer (Bruker, USA).

EDS spectra interpretation

Several overlaid EDS spectra are shown in Figures S11 and S9, where substrate spectra are included for comparison. Clearly, Ir is present in the deposited areas, albeit in seemingly low percentages; this is understandable given the considerable penetration depth of a 20 keV electron beam. By the Kanaya-Okayama electron penetration equation [8]:

$$R = 27.6 * E^{1.67} \frac{A}{\rho * Z^{0.89}} \quad [9]$$

where E = electron beam energy in keV, A = atomic weight in g/mol, ϱ = atomic density in g/cc, Z = average atomic number, and R = penetration depth in nm, a 20 keV beam's interaction volume would extend more than 3 μm into pure IrO_2. Films deposited per the routine shown in the experimental section were in the 50-100 nm range, as seen in the contact profilometry data in *Figure S2*, so EDS has clearly penetrated the whole of the thin film.

XPS Experimental Flow and Discussion

Three different XPS datasets were gathered. Given the possibility of surface variability due to light [10–12] and oxygen exposure, experiments were gathered both from replicates of identical polarization per session, as well as full sets of polarizations in a single session. After electrodeposition was performed via the above procedure, conditioning was performed on a sub-batch of 3-5 probes. Three sets of probes were brought to XPS: three probes per potential, coated and polarized at two potentials per day, in 200 mV increments between -0.2V and +0.8V; one probe per potential, coated and polarized all in one day, in 200 mV increments between -0.2V and +0.8V; and one probe per potential, coated and polarized all in one day, in 100 mV increments between +0.3V and +0.8V. The conditioning potential was chosen from 100-200 mV increments between -0.2 V and +0.8 V vs Ag/AgCl in 1 M NaCl, and applied for 180 s. The first experiment established instrument baselines for the probes and confirmed that XPS could measure changes in binding energy at the proposed scales. For the first dataset, XPS scans were performed on two polarizations per instrument session, for a total of three different instrument sessions (days) to cover the requisite polarization range, over a total of approximately 1 month. Second, a single session scanned all polarizations which had been batch fabricated on the same day; probes were stored in vacuum and away from light and fabrication-to-scan delays were kept to <= 24 h. In both cases, minimal alterations in species ratios or spectral lineshapes of the detail elemental spectra between -0.2 V and +0.2 V were visible but notable alterations began at +0.4 V, continued at +0.6 V, and appeared to cease at +0.8 V. Therefore, the third dataset was generated from a single session scanning all polarizations fabricated on the same day, but polarized in a narrow range around the transition voltage; these samples were polarized between 0.3 V and 0.8 V in 100 mV increments. The set included an unpolarized IrOx probe. One of the probes from each initial sub-batch was used for SEM/EDS, then XRD, then profilometry; if any probe failures were detected, another probe from the same sub-batch was used to conduct a repeated analysis.

At all steps of sample preparation, care was taken to control for contamination and air exposure, or at least normalize it across sample sets. The series of samples was prepared in consecutive operations from the same volumes of solution. After deposition and polarization, the sample batch was placed immediately in low vacuum and away from light (covered by aluminum foil) until they could be loaded in the XPS vacuum; this intermediate vacuum stage was typically 18 h and never more than 36 h. The sample batch was then mounted and loaded into XPS, being exposed to air no more than 30 min. Ultrahigh vacuum (UHV) pumping in the XPS cell took 2-4 h and sample measurements were begun immediately upon reaching target pressure levels. One additional dataset was used to discern Ir:O atomic ratios in an included figure: samples across a polarization range taken at multiple days in a ~2 month period; samples were prepared, loaded, and scanned in the same time frames as above but were not produced in consecutive deposition and polarization operations. This dataset is noted explicitly.

Although the preliminary and intermediate air contact raised the distinct possibility of environmental contamination (adventitious carbon and others), Ar+ ion etching was

not used on sample batches in this manuscript for two reasons: first, angle-resolved XPS (AR-XPS) scans of sample batches showed limited spectral differences with depth, within the penetration range of this technique (see Figure S6). Second, Ar+ ion etching caused severe reduction which did not appear to stabilize after a number of passes, suggesting the film is too sensitive for this technique (see Figure S7); some preferential sputtering is also indicated. Charging was not present in repeated scans of the sample batches (see Figure S8), so a flood gun was not applied.

AR-XPS Discussion

Angle resolved XPS (AR-XPS), as seen in Figure S6, yielded interesting information about the through-thickness composition of these samples; namely, that variation in spectra with depth is extremely subtle. Given the assumptions presented in the main text of this work, one logical conclusion is that the high level of porosity and low crystallinity of IrOx prepared in this manner provides a rapid enough proton diffusion through thickness via water molecules [13] for complete equilibration of the oxidation state. If we believe polarization mechanisms such as those presented in [14], polarization potentials induce inner sphere electron transfer across pi and sigma bonds, a fast and largely reversible process; even if speciation is reversed by ion contact and is some mixture of the slower concerted proton-electron transfer (CPET) and proton-coupled electron transfer (PCET) processes [15], they are still likely quick enough to take place through thickness in the minutes or hours the samples spend in air during preparation. Further investigation is needed to elucidate transient mechanisms, possibly in-situ or in more controlled ion application.

XPS Fitting Procedure

Fitting assignments were necessary for quantitative trends. As discussed in supplementary material, IrOx's varying semiconductor/metallic behavior [3,4] precluded work function charge referencing [5]; spectra are presented as-measured, or AdC referenced where explicitly noted. After referencing, the analysis was guided by raw structure in order to balance multiple aims: simultaneous fitting of multiple spectra; coupled position and shape analysis of O 1s against Ir 4f, valence band spectra, and potentially overlapping contaminants (Na, Cl, C, etc.); ratios of Ir 4f / O 1s fit species ratios; ratios of Ir to O generally; and work functions (He I cutoff; Figure S15). Scofield cross sections were used as presented in software (Table S2), across all major core levels apparent in survey spectra.

Background (Shirley) subtracted raw data and 1st/2nd differentials for O 1s and Ir 4f are presented in Figure S16; multiple local minima/maxima were observed. Assuming alterations in O 1s would be mirrored in Ir 4f, emphasis was placed on extracting quantitative trends from O 1s, to transfer to Ir 4f. Figure S16 guided O 1s placement of hydroxide and water components typical to literature, in addition to a small Na 1s Auger-Meitner line [16–20], all as Gaussian/Lorentzian (GL) curves with roughly 30% instrumental widening. Simultaneous fit of several low polarization potentials (chosen based on Figure 3) yielded average positions, which were then locked in order to identify residuals; inspection suggested a fourth component al low B.E., tentatively labeled as oxide. These components were applied to all potentials and simultaneously fit for self-consistency. Figure 6a,c,e and Table S2 show hydroxide:oxide trends and curve parameters extracted to be applied to Ir 4f fits. Single spin-orbit split doublet fits (tentatively hydroxide) of Ir 4f, with 5p ½ contributions [21], yielded significant high B.E. residuals across potentials; after similar position averaging to O 1s, component positions were locked, a second doublet was inserted at high B.E. (tentatively oxide), and simultaneous fitting was performed across all potentials. Reasonable self-consistency now existed: both O 1s and Ir 4f featured two species, both the lower B.E. O 1s species and higher B.E. Ir 4f species followed similar polarization trends, O 1s and Ir 4f components featured similar full-width-at-half-maximums (FWHM), and both featured species of the same lineshape. Based on the aforementioned possible semiconductor behavior, as well as inclusion in mechanistically-underpinned studies, satellite doublets were added, with estimated derived manually in Fityk software (Table S1); satellite fitting justification is discussed in more detail below. Finally,

high B.E. Ir 4f and low B.E. O 1s species were made inwardly asymmetric using the LF mathematical function, physically interpreted to be accounting for small secondary effect features difficult to justify individually. Doniach-Sunjin lineshapes are common with metal oxides but the LF function features simpler implementation and more granular (i.e. physically meaningful and defensible) parameter control. Final Ir 4f fits, parameters, and extracted trends are shown in Figure 6b,d,f and Table S2.

Several assumptions were made, and justifications required, during this process. First, Ir 4f "oxide" component B.E. shift direction was chosen based on the general assumption that increased potential increases ligand oxidation [14,22,23]. Also, raw Ir 4f spectra increased at higher polarizations which are generally assumed to produce the more anhydrous and crystalline IrO_2. General Ir complexes have been known to show high B.E. Ir 4f shifts with increased complexation [24]. Doublet positions may have been swapped based on an alternative mechanistic understanding that defects were taking place [25,26], though the material studied differed and some critique has been published about the completeness of the analysis [21]. Second, satellite trends and starting values were drawn from both literature and valence analysis. Major valence band features in Figures 3e and S17 trend towards E_F with increased potential (alternatively, E_F trends towards these features). Minimal GL fitting to UPS He II, UPS He I, and XPS valence (Figure 5a,c,e), although being more qualitative than physically meaningful, yielded extracted area ratios (Figure 5b,d,f) which demonstrated transfer of intensity distribution to features farther from E_F; this was interpreted as Ir 4f satellite features trending closer to main features, being small by comparison (by the $1/\epsilon^2$ relation), and being obscured to some degree by increasing Ir 4f doublet asymmetry. The interfering nature of the first and third statements necessitated constant satellite position offsets and area ratios, as well as a small amount of parameter variation between individual potentials. Satellite starting positions cannot be extracted directly from valence spectra, but were instead derived from EELS plasmon peaks for IrO_2 [27] and PDOS simulations [21,25,26,28] (1-2 eV); EELS plasmon peaks are typically correlated to satellite offset [29–31]. Seeding Fityk with these values yielded ~Δ1.2 eV and 12-18% area ratios (Table S1). Ir:O ratios (discussed below) and O 1s / Ir 4f species ratio support satellite assignment and are consistent to each other.

Supplementary Data

Table S1. Satellite parameters derived from simulation of Ir 4f peak fitting.

Test Name	dS (eV)	AS (ratio)
**Ir 4f_1**	0.9	0.2017
**…**	1.0	0.1654
**…**	1.1	0.1381
**…**	1.2	0.1173
**…**	1.3	0.1015
**Ir 4f_2**	0.9	0.2136
**…**	1.0	0.1743
**…**	1.1	0.1447
**…**	1.2	0.1221
**…**	1.3	0.1049
**Ir 4f_3**	0.9	0.1540
**…**	1.0	0.1245
**…**	1.1	0.1023
**…**	1.2	0.0855
**…**	1.3	0.0730

Table footnotes: key is as follows. dS = satellite separation from doublet peak, in the range of ~1-1.2 eV by residual inspection. AS = satellite area ratio as compared to main peak, range as estimated from. Parameters not shown: dF = spin orbit splitting of Ir 4f, averaged from database values [32] to 2.98 eV and held at that value for all runs. dP = spin orbit splitting of Ir 5p; in the 15 eV range based on [21], empirically averaged to 15.43 eV based on lowest residual initial fits and held at that value for all runs.

Table S2. Fit parameters for components shown in Figure 6.

Transition	Index	Name	RSF	Line Shape	Area Constraint	FWHM	FWHM Constraint	Position	Position Constraint
**Ir 4f**	A	Hydroxide 7/2	5.021	GL(30)	None	0.75	0.2, 2.5	61.97	69.8, 58.5
	B	Hydroxide 5/2	5.021	GL(30)	A*0.75	0.75	A*1	64.97	A+3
	C	5p 3/2	0	GL(30)	None	4.36	0.25, 6	49.59	49.6, 49.4
	D	5p 1/2	0	GL(30)	C*0.5	4.36	C*1	64.59	C+15
	E	Hydroxide 7/2 sat.	5.021	GL(30)	A*0.25	2.32	0.5, 3	62.97	A+1
	F	Hydroxide 5/2 sat.	5.021	GL(30)	B*0.25	2.32	E*1	65.97	B+1
	G	Oxide 7/2	5.021	LF(0.5,1.5,20,200)	A*8.49	1.65	1, 2.5	62.27	A+0.3
	H	Oxide 5/2	5.021	LF(0.5,1.5,20,200)	G*0.75	1.65	G*1	65.27	G+3
	I	Oxide 7/2 sat.	5.021	LF(0.5,1.5,20,200)	G*0.25	2.32	E*1	63.27	G+1
	J	Oxide 5/2 sat.	5.021	LF(0.5,1.5,20,200)	H*0.75	2.32	E*1	66.27	H+1
**O 1s**	A	Hydroxide	0.78	GL(30)	None	1.8	0.4, 9	531.64	Locked
	B	Na Auger	0.78	GL(30)	None	1.8	A*1	534.7	Locked
	C	Water	0.78	GL(30)	None	1.8	A*1	533.7	Locked
	D	Oxide	0.78	LF(0.8,1.3,10,500)	None	2.12	0.5, 2.5	530.7	531.4, 527.8

Table footnotes: RSF is relative sensitivity factor, otherwise known as Scofield cross section. FWHM is full-width at half-maximum. All parameters shown above are exactly as input into CasaXPS software.

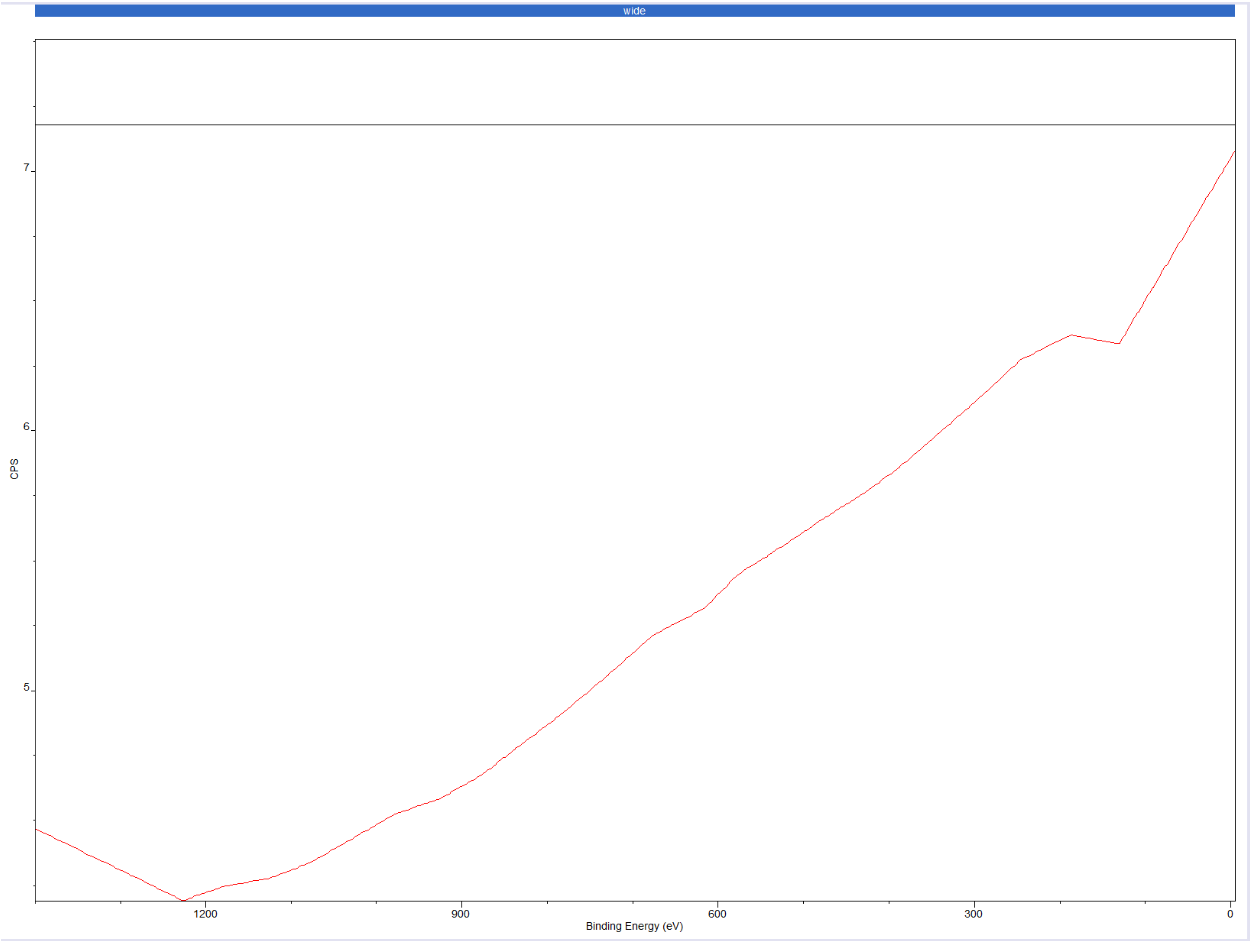

Figure S1. Transmission function for data pictured in main text.

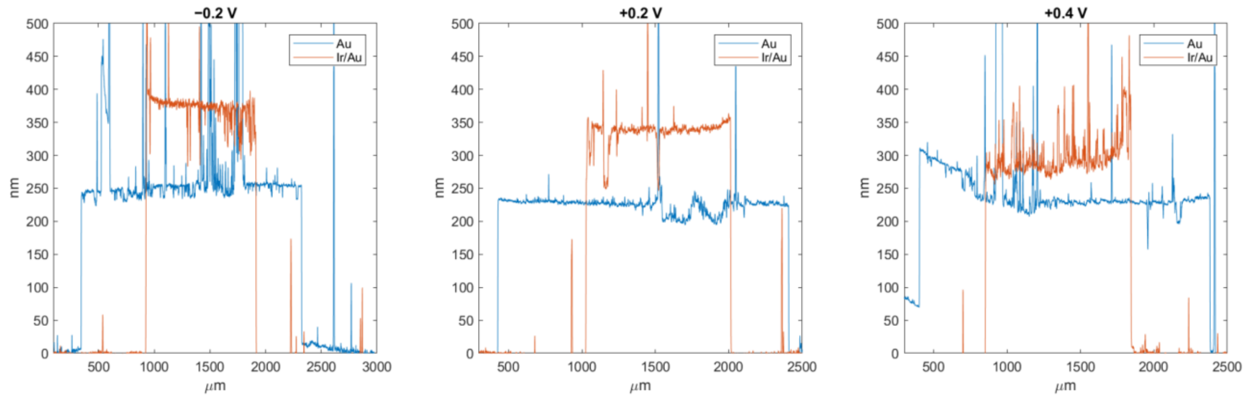

Figure S2. Example contact profilometry, three samples. Some adhesive compression present in scan of +0.4 V sample, accounted for by static data offset. While final IrOx thicknesses had some variation, film layers were rarely thinner than ~50 nm and often closer to 100+ nm. Data serves as reference for EDS Kanaya-Okayama calculation.

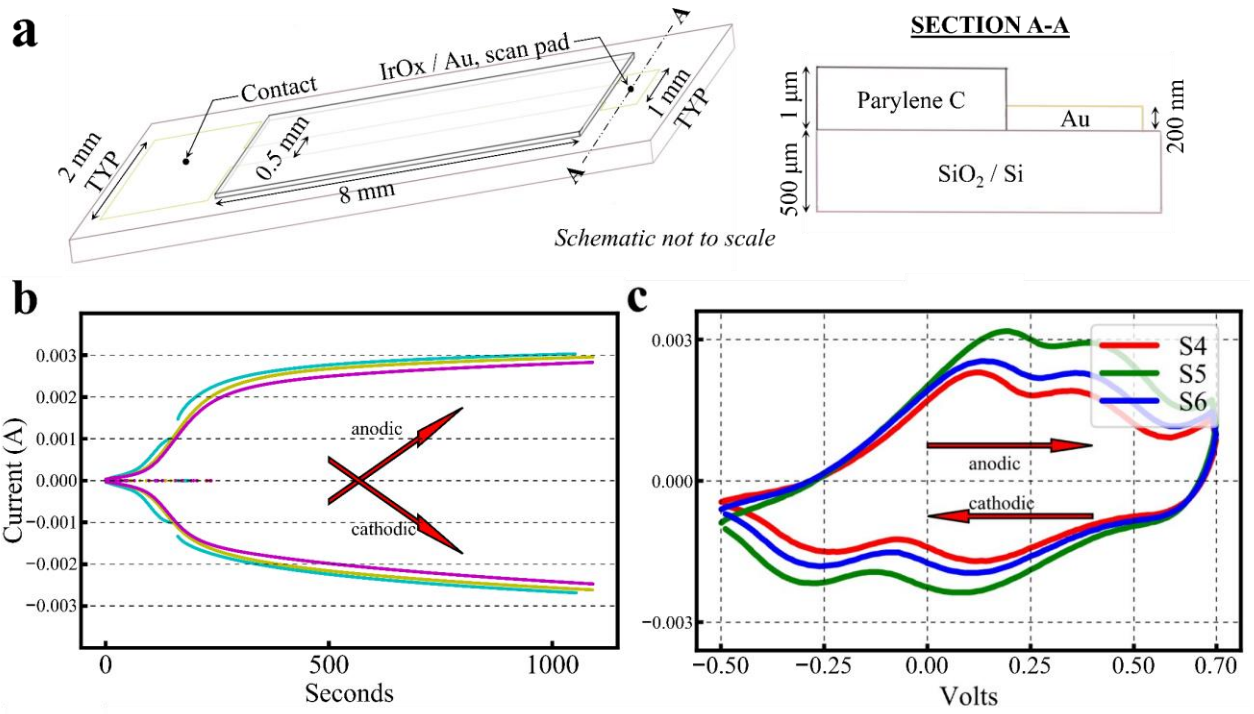

Figure S3. a) Isometric and section view of substrate used for coating and analysis; see **Figure S2** for typical IrOx thickness. **b**) Amperogram of pulsed potential electrodeposition of samples S1, S2, and S3. Discontinuity in S3's curve resulted from device setup error. **c**) Voltammograms of cyclic voltammetry electrodeposition of samples S4, S5, and S6, as shown by final cycle; these three samples were produced in sequence in a single session. Outside of subfigure **b**), all samples analyzed in this study were produced via this cyclic voltammetry strategy.

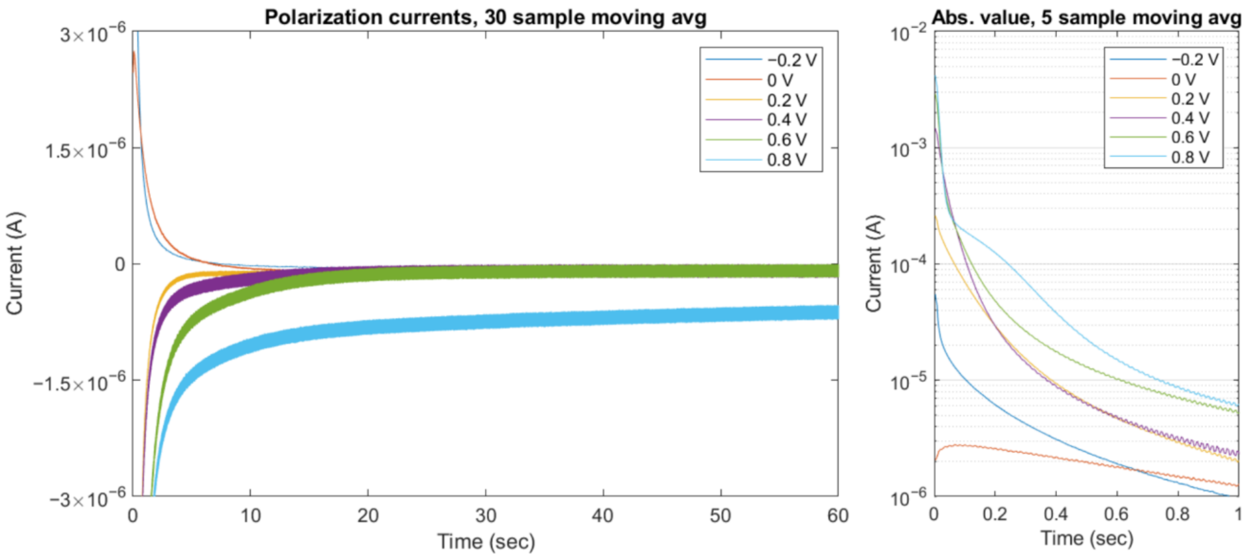

Figure S4. Typical polarization stage currents. Main chart shows polarization current over the first 60 s, with side panel focusing on the first second; in the side panel, all currents are taken as an absolute value and plotted against a log scale. In both cases, oscillations are result of low resolution amplifier electronics to prevent current overflow during first 100 ms; no stirring was performed during the polarization process, but no vibration isolation was applied.

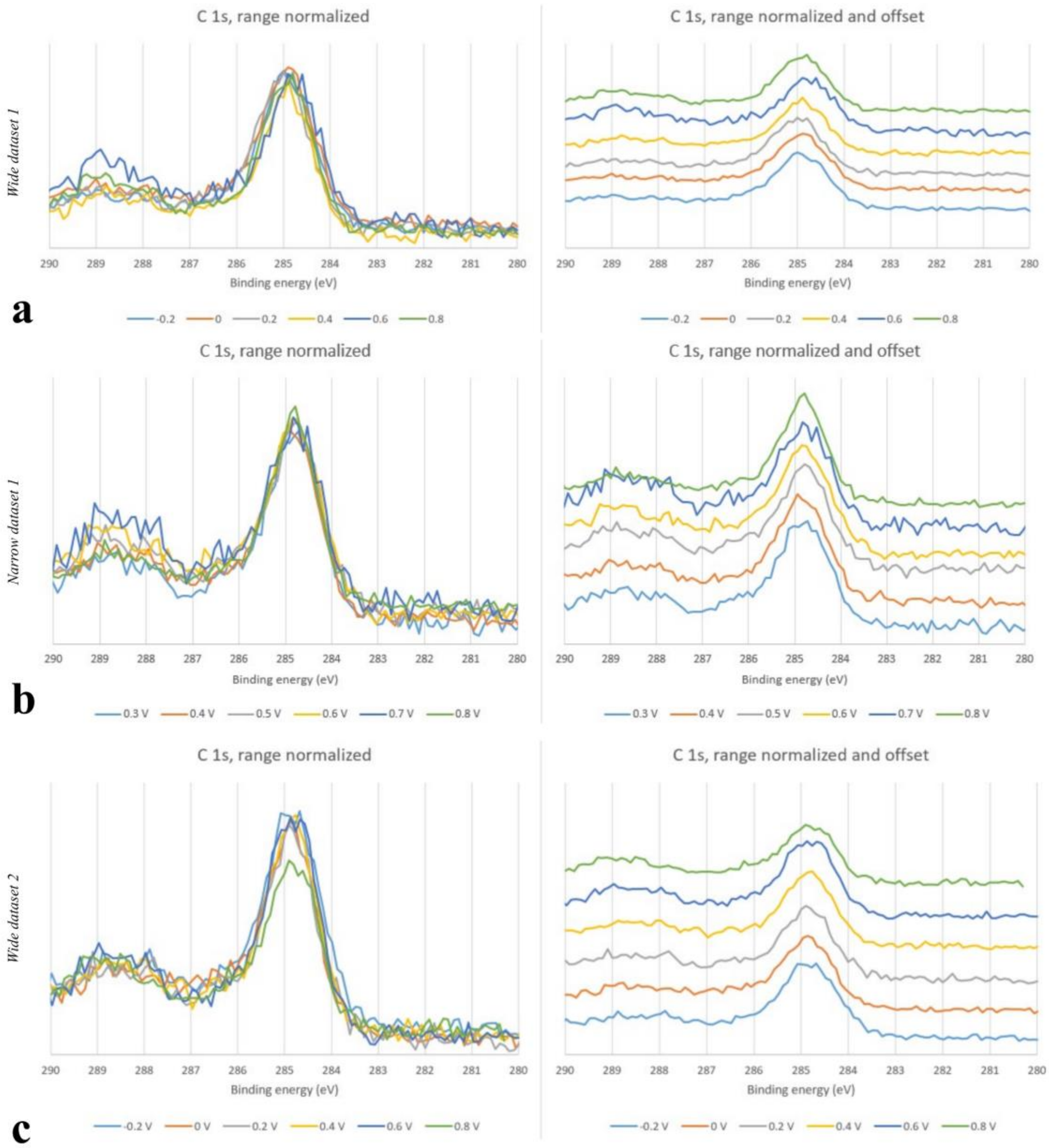

Figure S5. C 1s detail spectra, all polarizations. **a**) single-day, wide voltage range dataset. **b**) single-day, narrow voltage range dataset. **c**) one month, wide voltage range dataset. Stacks confirm relative consistency of binding energy scale for spectral comparison in main paper figures in the absence of explicit charge referencing, given that sample batches were exposed to internally identical conditions.

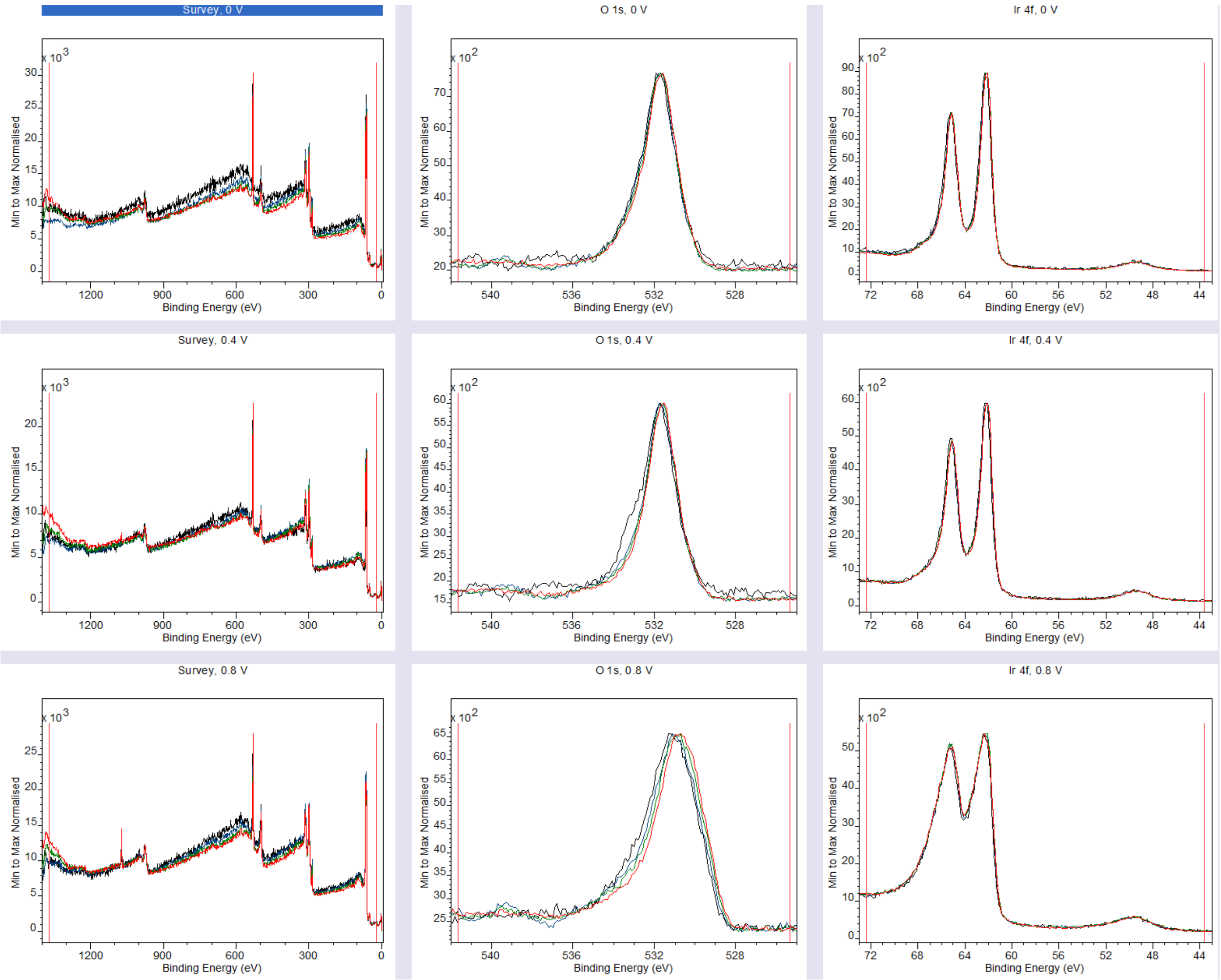

Figure S6. Survey, O 1s, and Ir 4f scans at different polarizations for different all AR-XPS angles. Transition and voltage are called out at each title block. Color key is as follows: red = 0 degrees, green = 40 degrees, blue = 55 degrees, and black = 70 degrees.

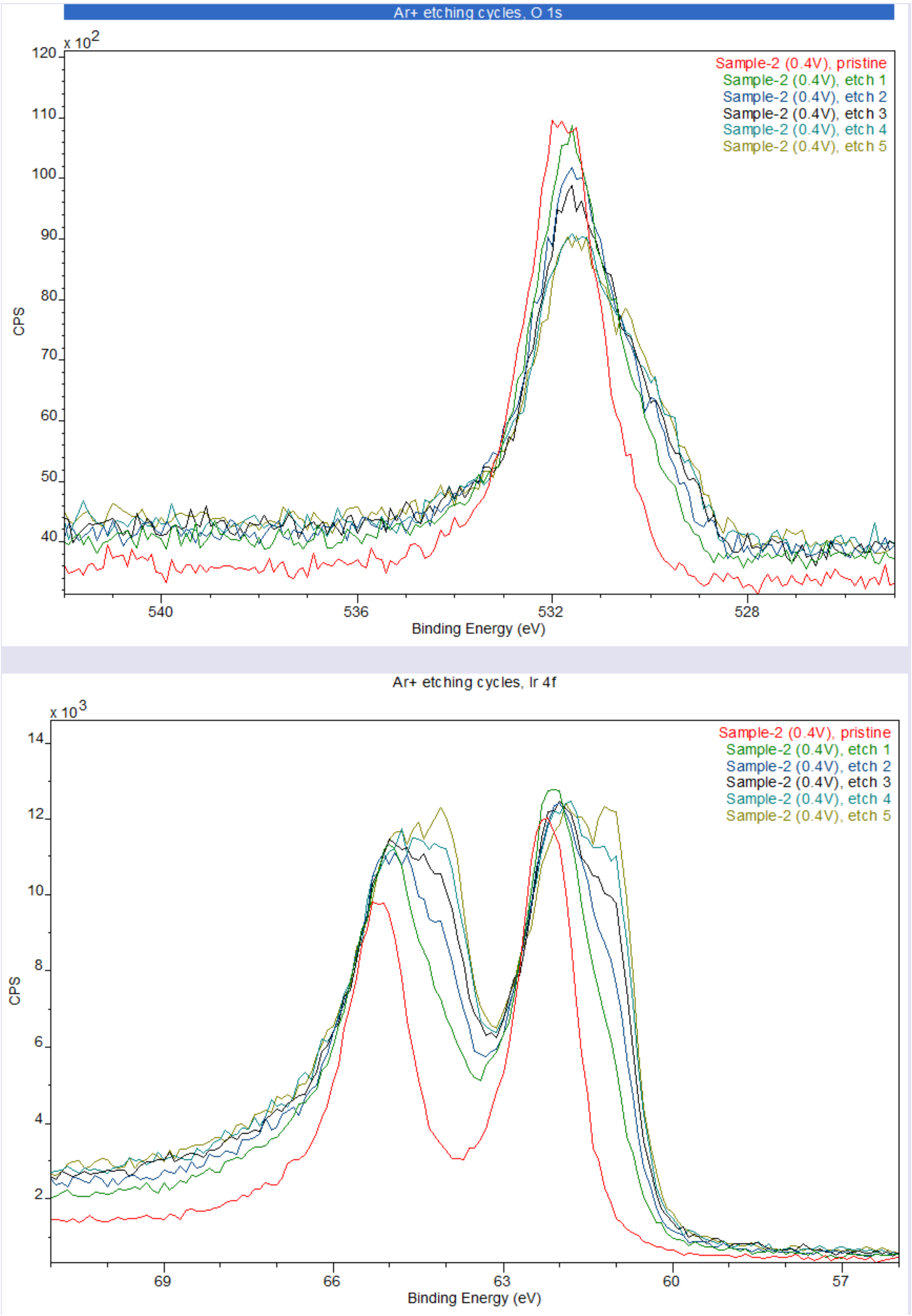

Figure S7. Ar+ ion etching results from polarized IrOx thin film.

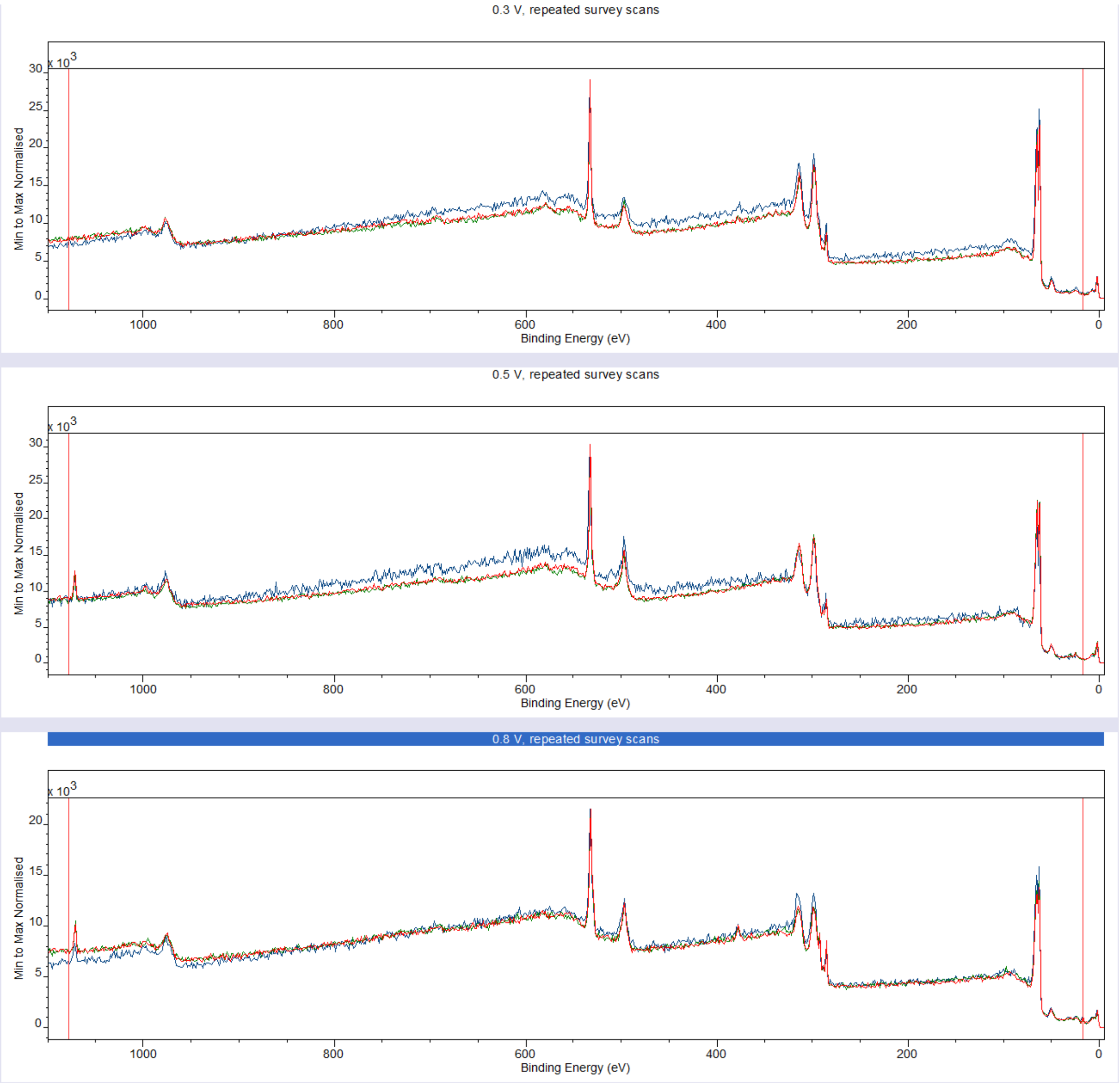

Figure S8. Lack of evidence for sample charging for films polarized at multiple voltages, as listed in title blocks. Colors indicate repeated scans.

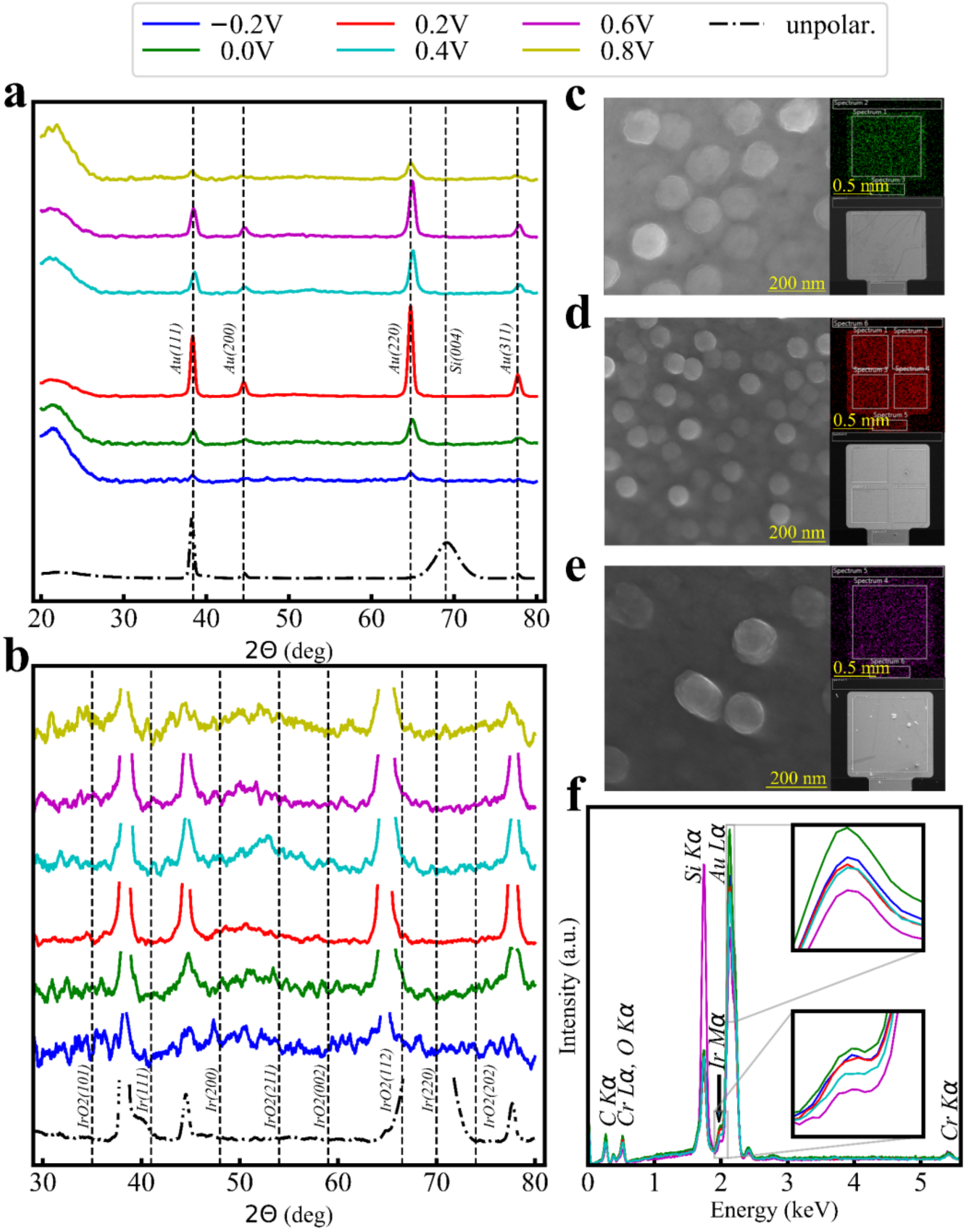

Figure S9. Full scale (**a**) and baseline (**b**, 10% of full amplitude scale) XRD with all PC potentials stacked, including an unpolarized probe included for reference. SubFigure **a)** features dashed lines for visual emphasis of identified crystalline peaks, while **b**) provides indicators for phases of a crystalline IrO_2 film, as retrieved from [33]. **c-e**) Visual morphological inspection at the nanoscale; SEM images (both nano- and macroscale), and EDS maps for Ir, for potentials of -0.2 V, +0.2 V, and +0.6 V, respectively. **f**) Bulk film elemental data, in overlaid EDS spectra for all polarizations. Zoomed insets are provided for the the Ir M*α* and Au L*α* peaks to demonstrate relative peak intensities with polarization; although trending may be apparent, intensity cannot be taken as an absolute, given variable film thickness (i.e. variable interaction volume) and scale normalization. Instead, intensity groupings should be taken as qualitative evidence of similar relative amounts. All XRD figures have had polynomial fit background subtraction performed via the Zhang library [34], followed by unity-based normalization for 2θ's of 30-80 degrees, and smoothed by a 1-D pixel moving average filter of window size 10. Raw XRD data is available in supplemental **Figure S10**. Even though signal returns of the thin films were favored by the shallow interaction depth of GI-XRD (see methods section), only substrate Miller indices could be definitively identified.

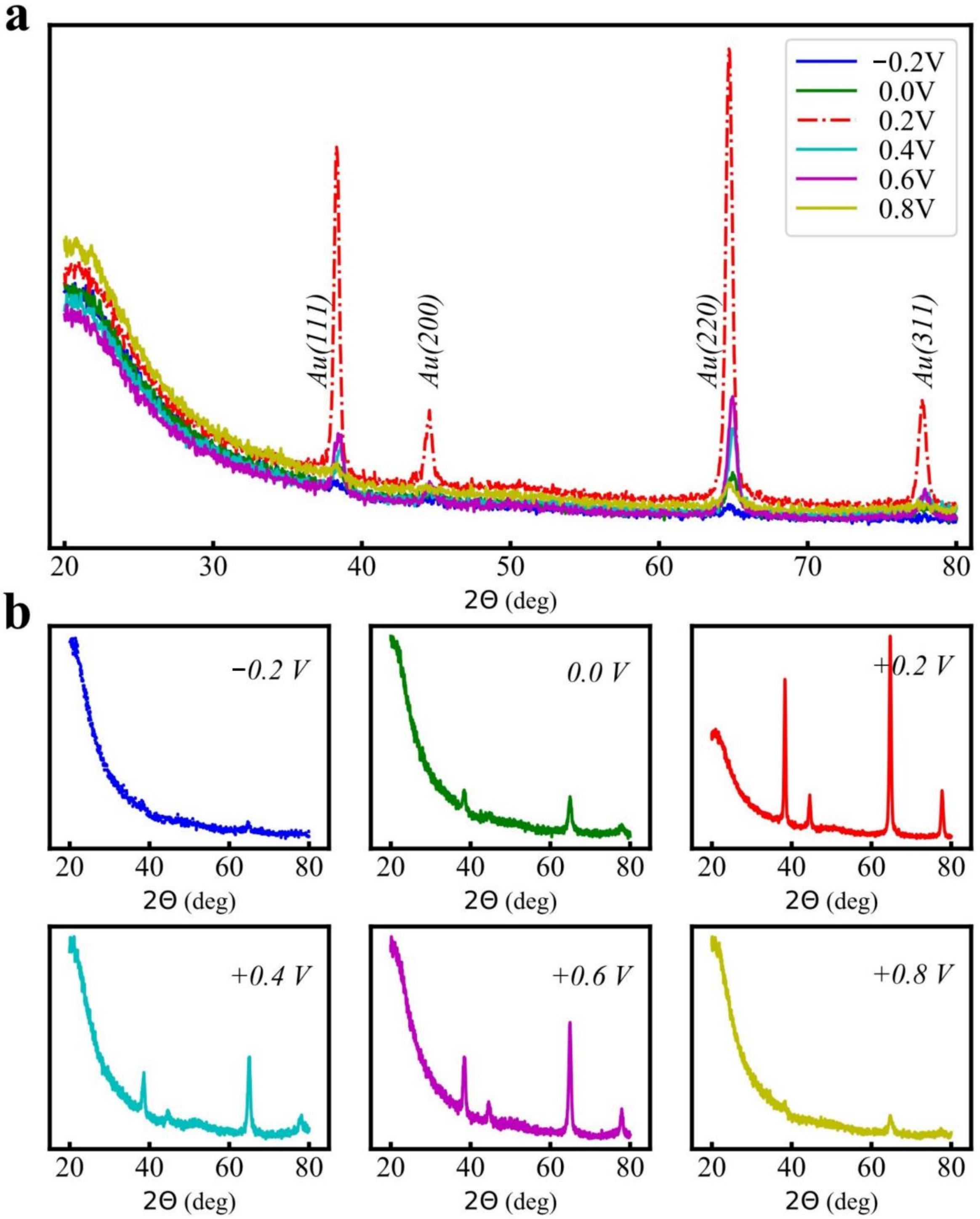

Figure S10. Raw XRD data across a full sample set. a) all polarizations overlaid, with the 0.2 V featuring a dashed line to highlight it. b) all polarizations, separated. Demonstrates raw version of Figure S9 prior to background subtraction.

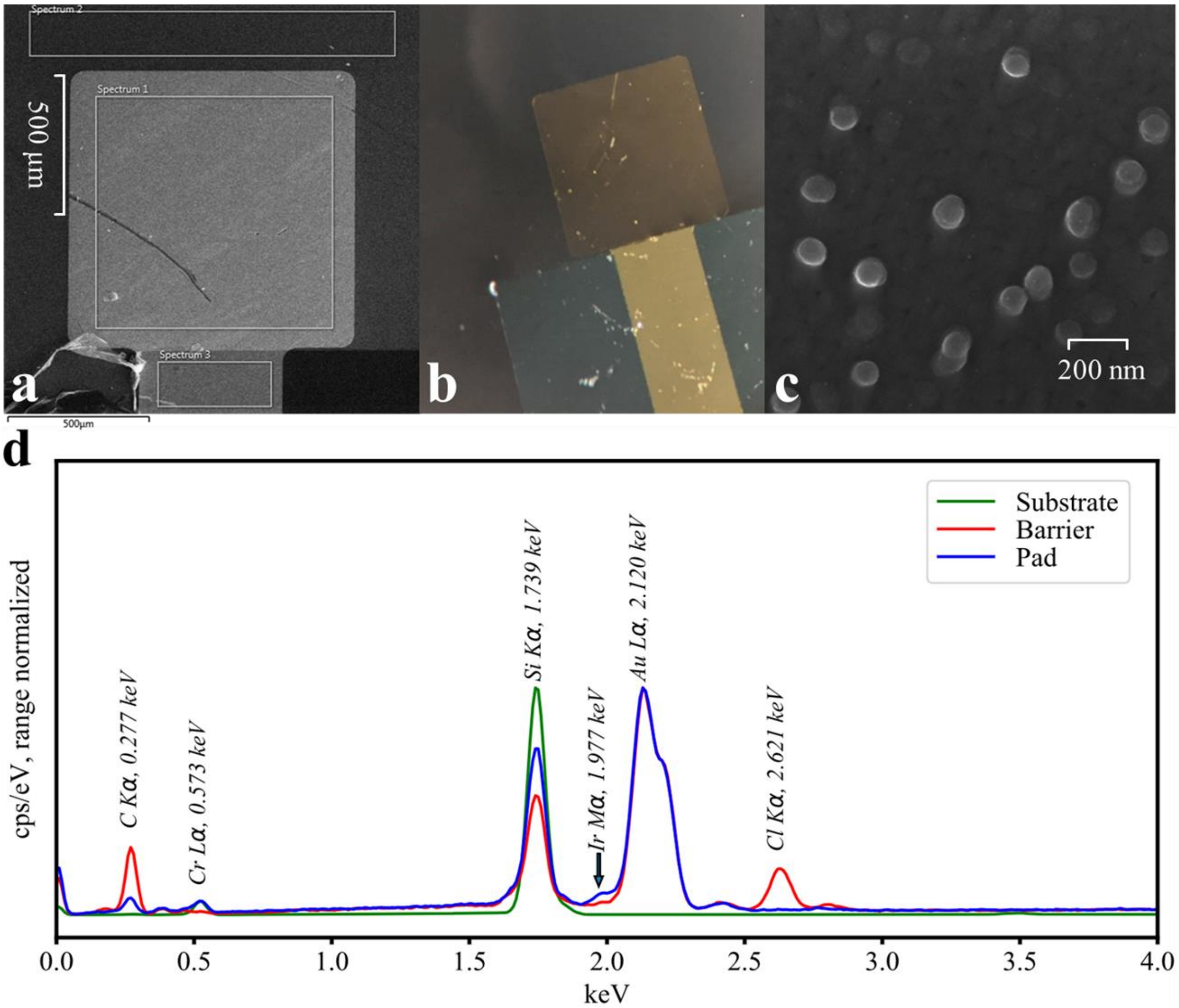

Figure S11. Imagery and EDS spectra of typical electrodeposited and unpolarized IrOx probe, demonstrating localization of deposited film. a) SEM image of substrate surface and thin film, with EDS area scan boxes (see spectra in d)); spectra 1, 2, and 3 cover the IrOx / Au / Cr pad, SiO_2 / Si substrate, and Parylene-C barrier atop Au / Cr, respectively. b) Optical image of substrate. Note translucence of Parylene-C barrier film and color transition from yellow Au trace to brown IrOx thin film. c) Nanoscale morphology of typical surface, taken at 25 kV, a working distance of 5.44 mm, and a magnification of 295 kx. d) EDS spectra for three scan boxes overlayed onto a); Ir percent on pad appears to be relatively low for two main reasons: high electron penetration depth (Kanaya-Okayama range estimate is upwards of 3 micron) and range normalization of all spectra.

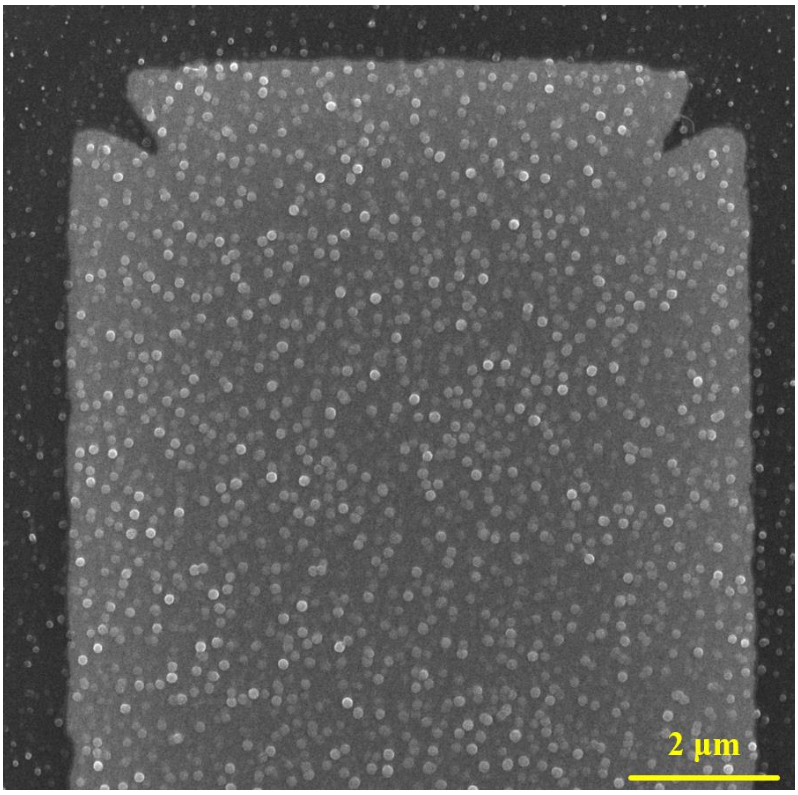

Figure S12. SEM image of IrOx film with no conditioning step applied. Note similarity to polarized films shown in **Figure S9**.

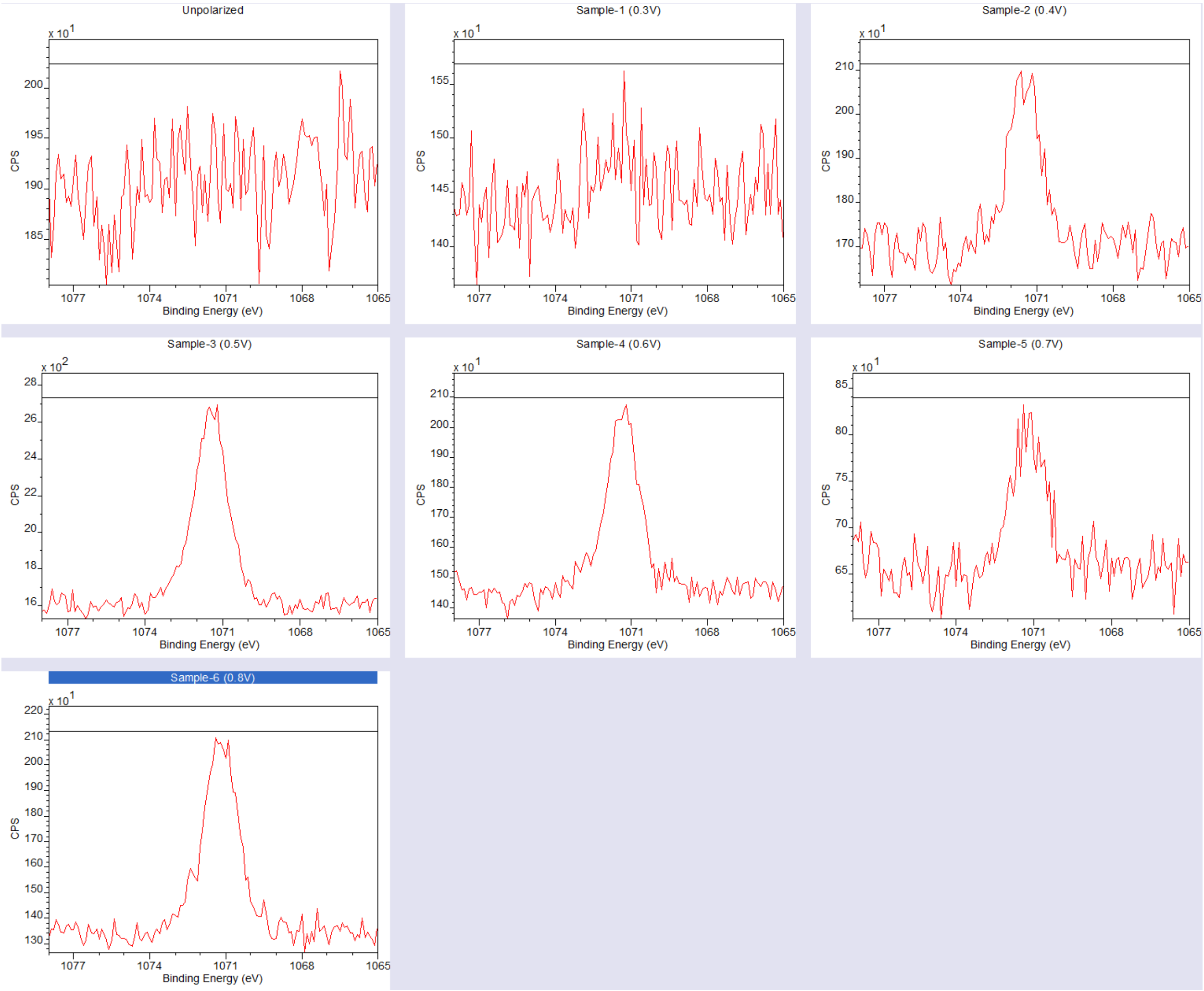

Figure S13. Na 1s presence in core orbital surveys of polarized films. Voltages listed in title blocks.

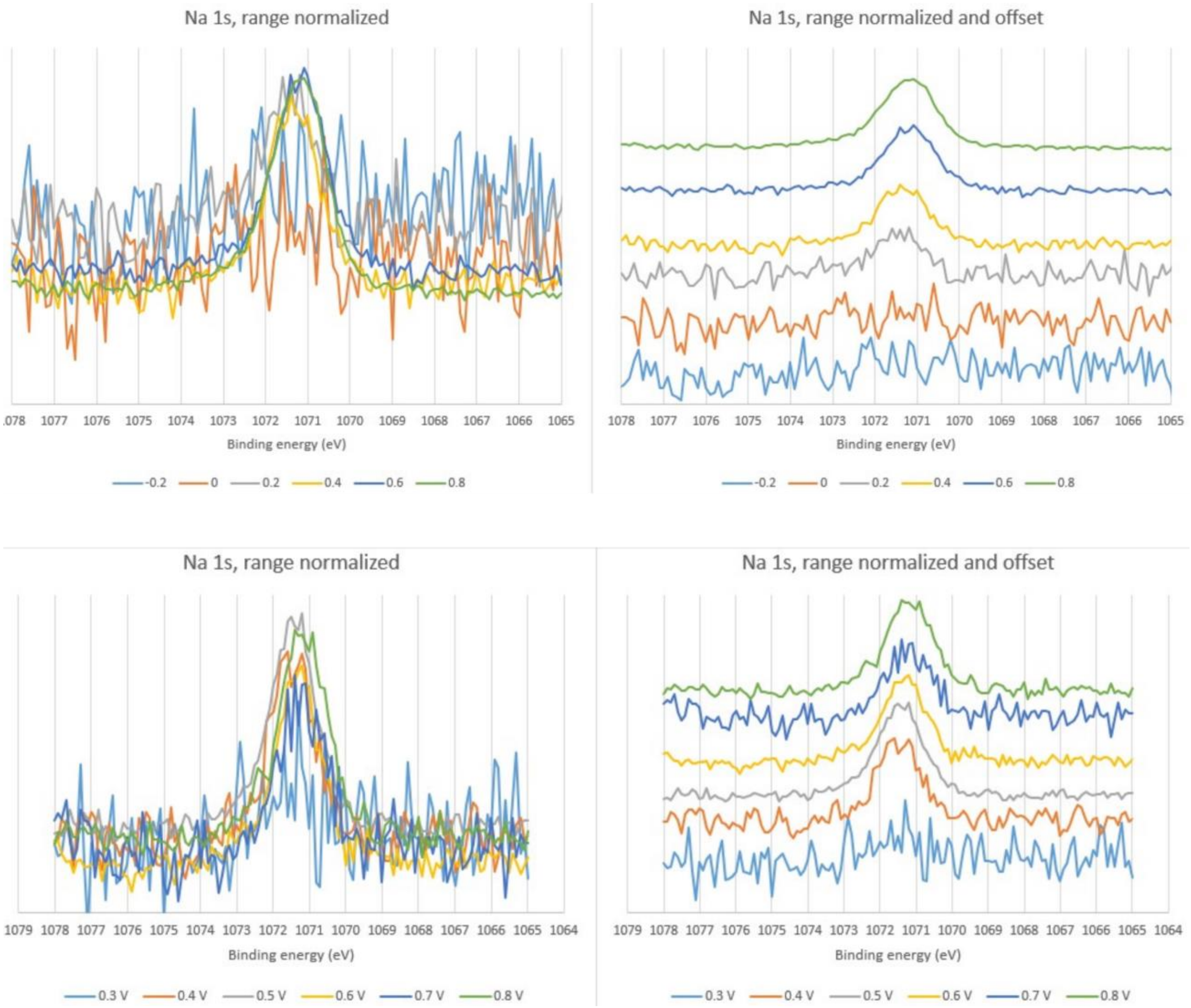

Figure S14. Na 1s detail spectra, all polarizations. **a**) Normalized and offset plots for single-day, wide voltage range dataset. **b**) Normalized and offset plots for single-day, narrow voltage range dataset. Trends support the notion that PBS polarization induces some Na surface bonding but that position (i.e. Auger-Meitner position) are highly stable and interference with spectral trends is limited.

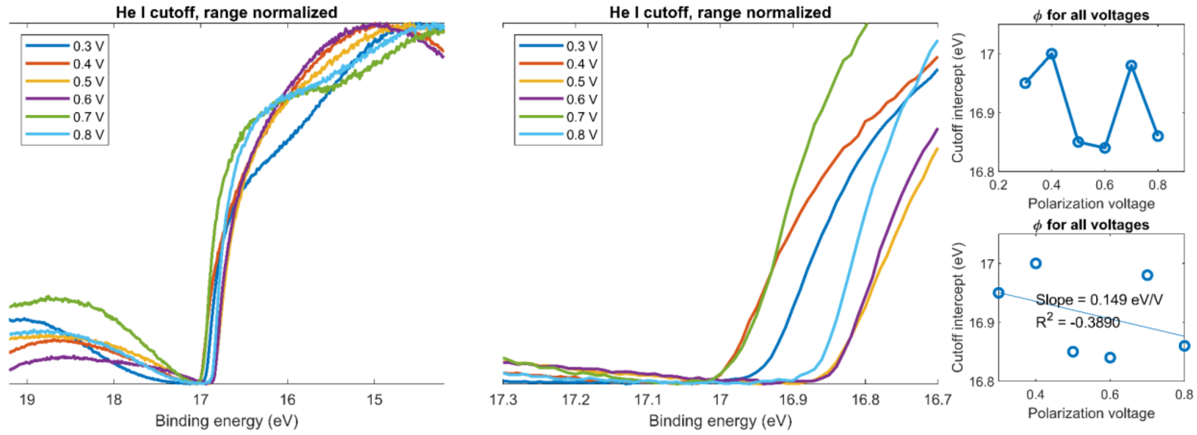

Figure S15. Work function measurements by He I cutoff, all polarizations. **a**) Data, range normalized. **b**) Range normalized data inspected at background intercept. **c**) Intercept plotted, connection line. **d**) Intercepts plotted as scatter, least squares linear regression overlay. Pearson coefficient indicates significant nonlinearity.

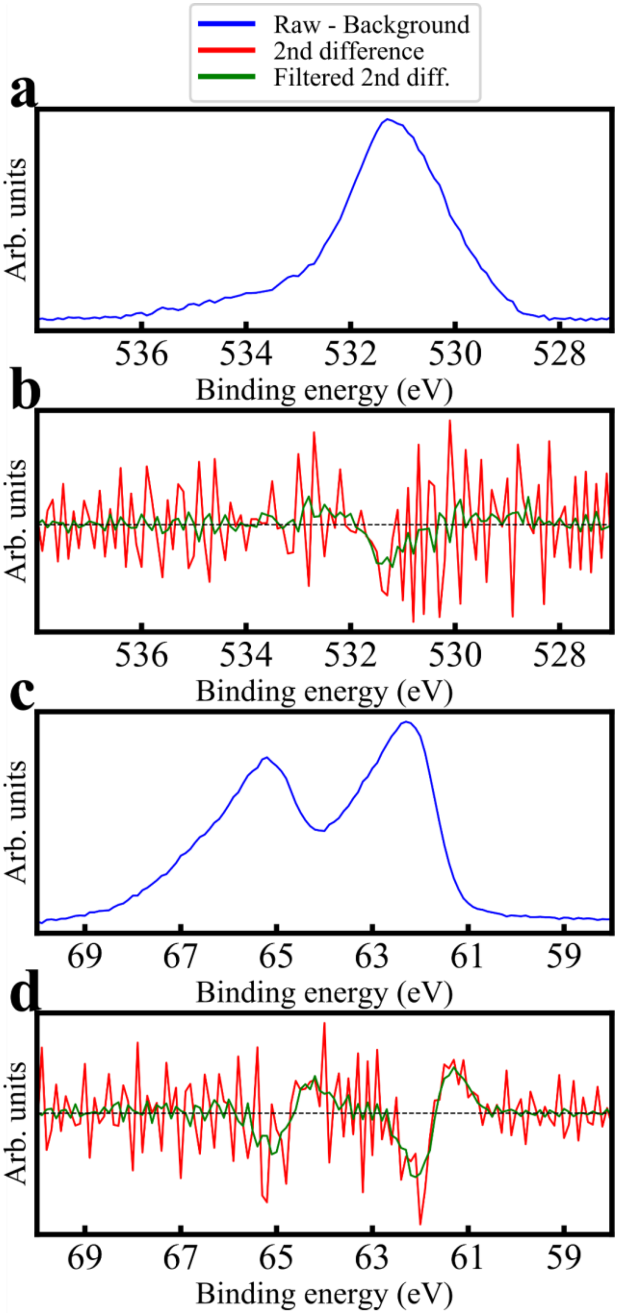

Figure S16. Demonstration of initial feature locations as observed in raw data. Raw data with Shirley background subtraction for O 1s and Ir 4f are shown in a) and c), respectively. Second differential estimates, as calculated by the forward finite difference method, are shown for O 1s and Ir 4f in b) and d), respectively. Clearer overlays have been provided by 1-D moving average filter.

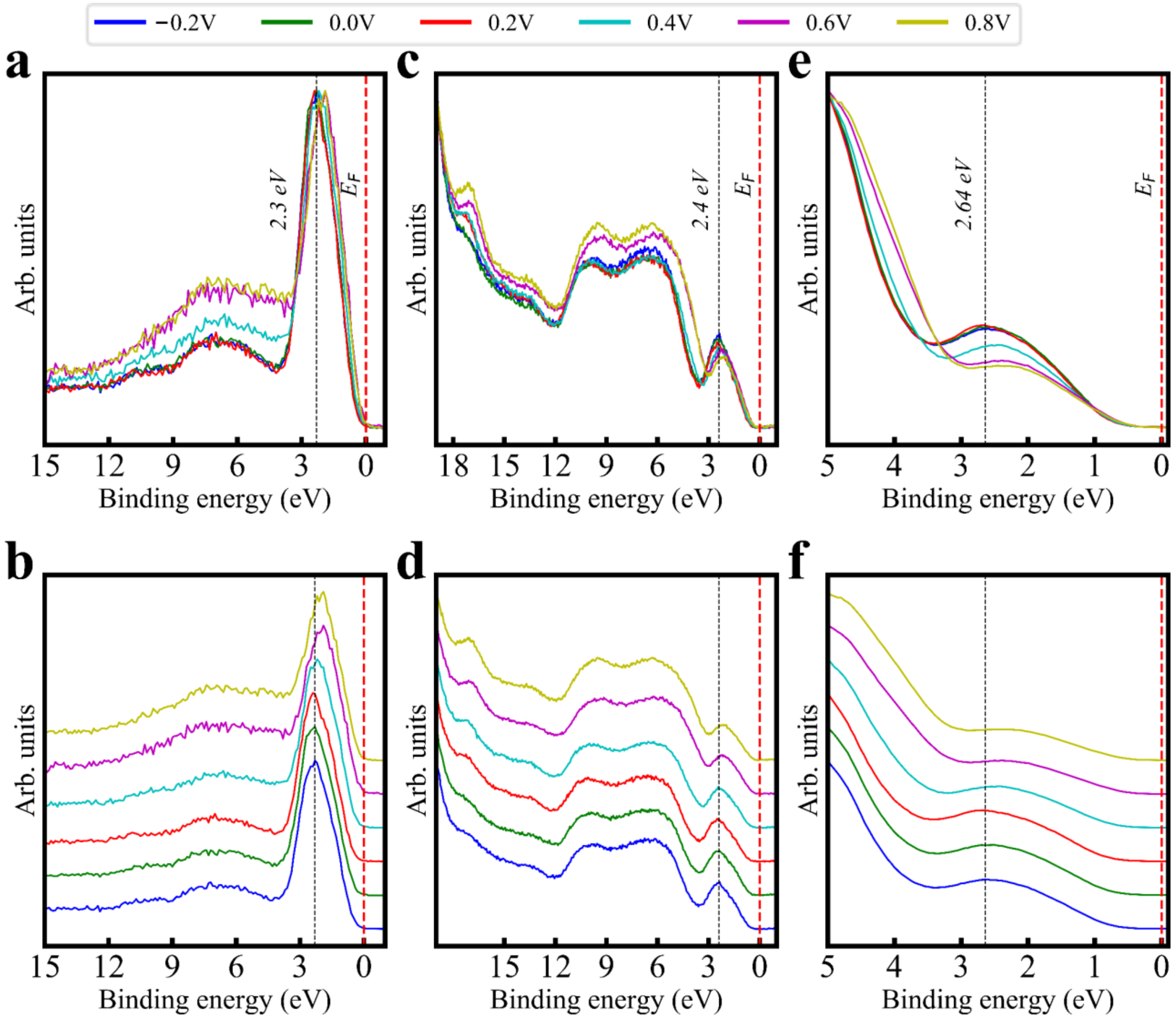

Figure S17. a) and **b**) overlaid and stacked detail spectra for XPS valence (1486.6 eV), across polarization range. **c**) and **d**) overlaid and stacked spectra for He II valence (40.8 eV), across polarization range. **e**) and **f**) overlaid and stacked spectra for He I valence (21.2 eV), across polarization range.

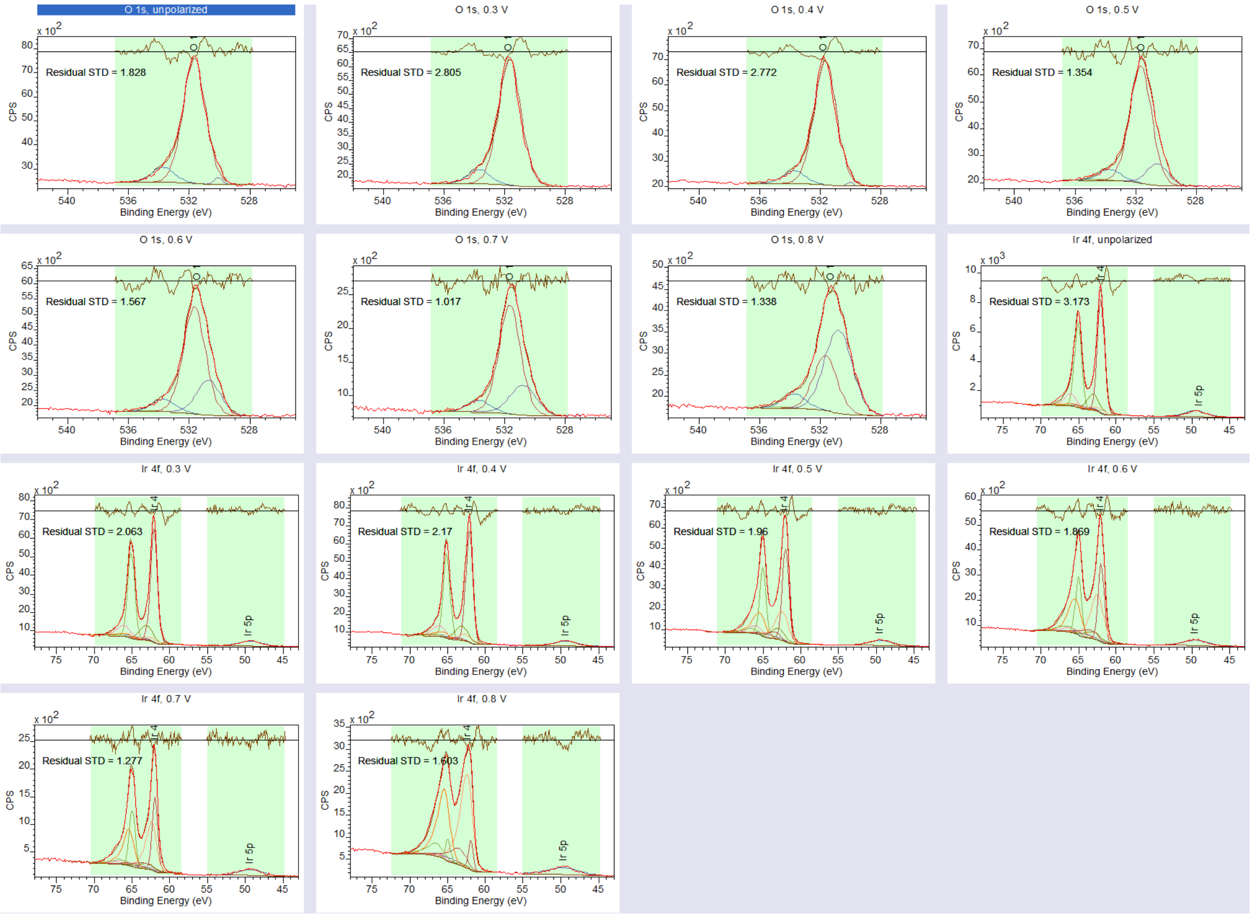

Figure S18. fits for all polarizations, as used to derive quantities shown in main text **Figure 6**. Transitions and polarization voltages can be found in title blocks.

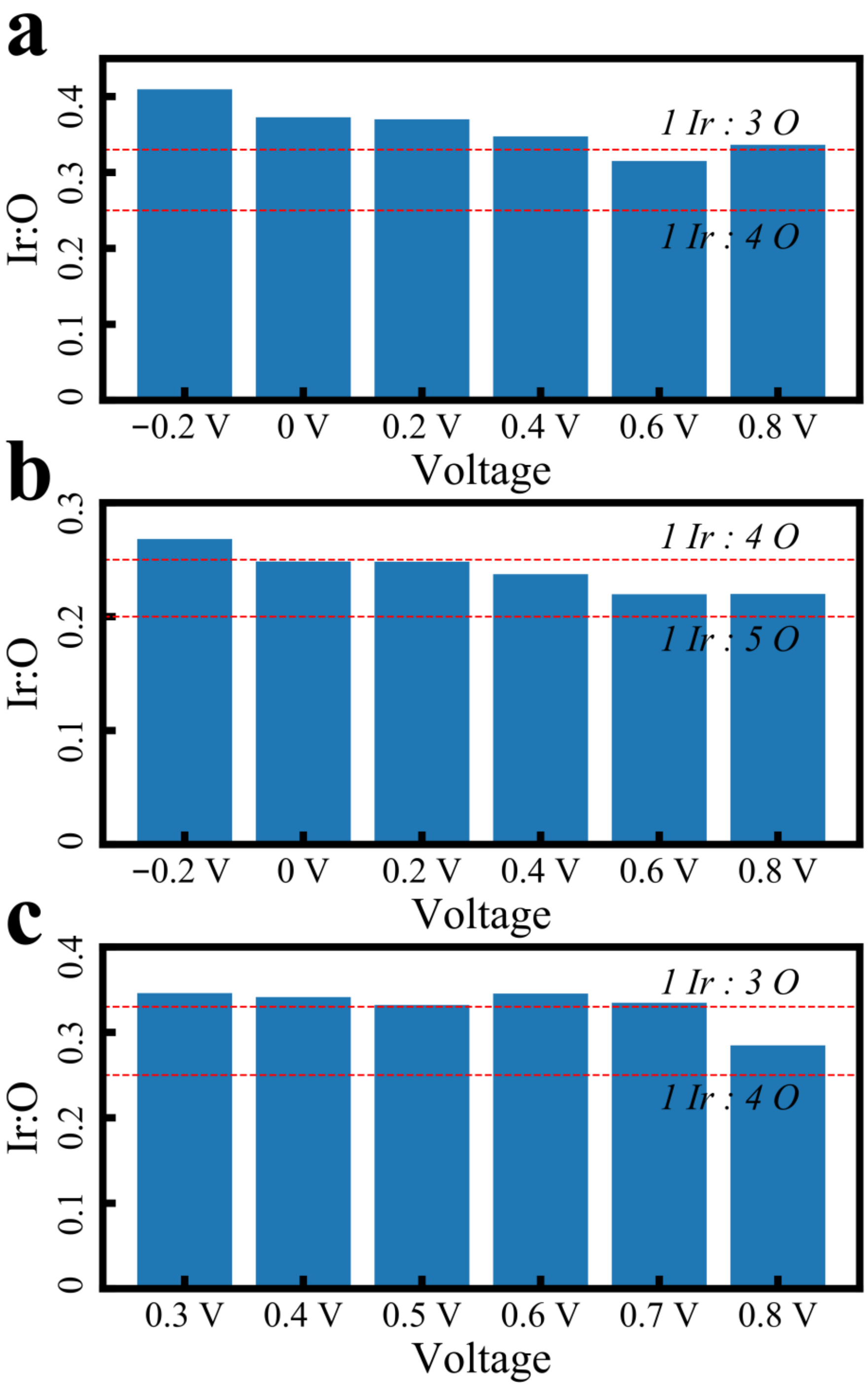

Figure S19. Ir-O ratios, as drawn from region areas of survey scans. Spectra from **b**) are available in the main text. Spectra underlying **a**) and **c**) were gathered separately. Spectra underlying **a**) is the non-consecutive dataset called out in Experimental section.

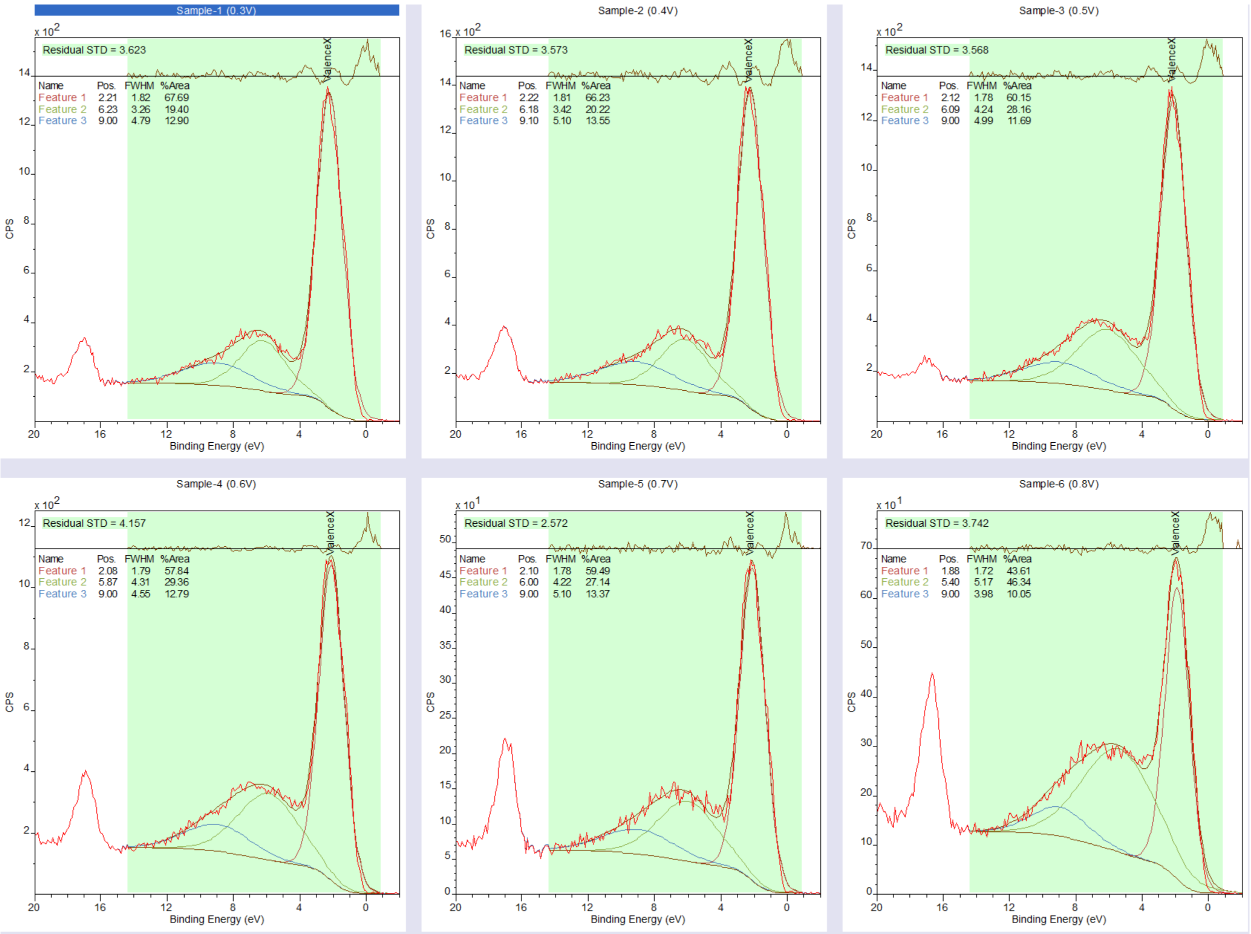

Figure S20. XPS valence rough fits across the polarization range. Voltages can be found in title blocks.

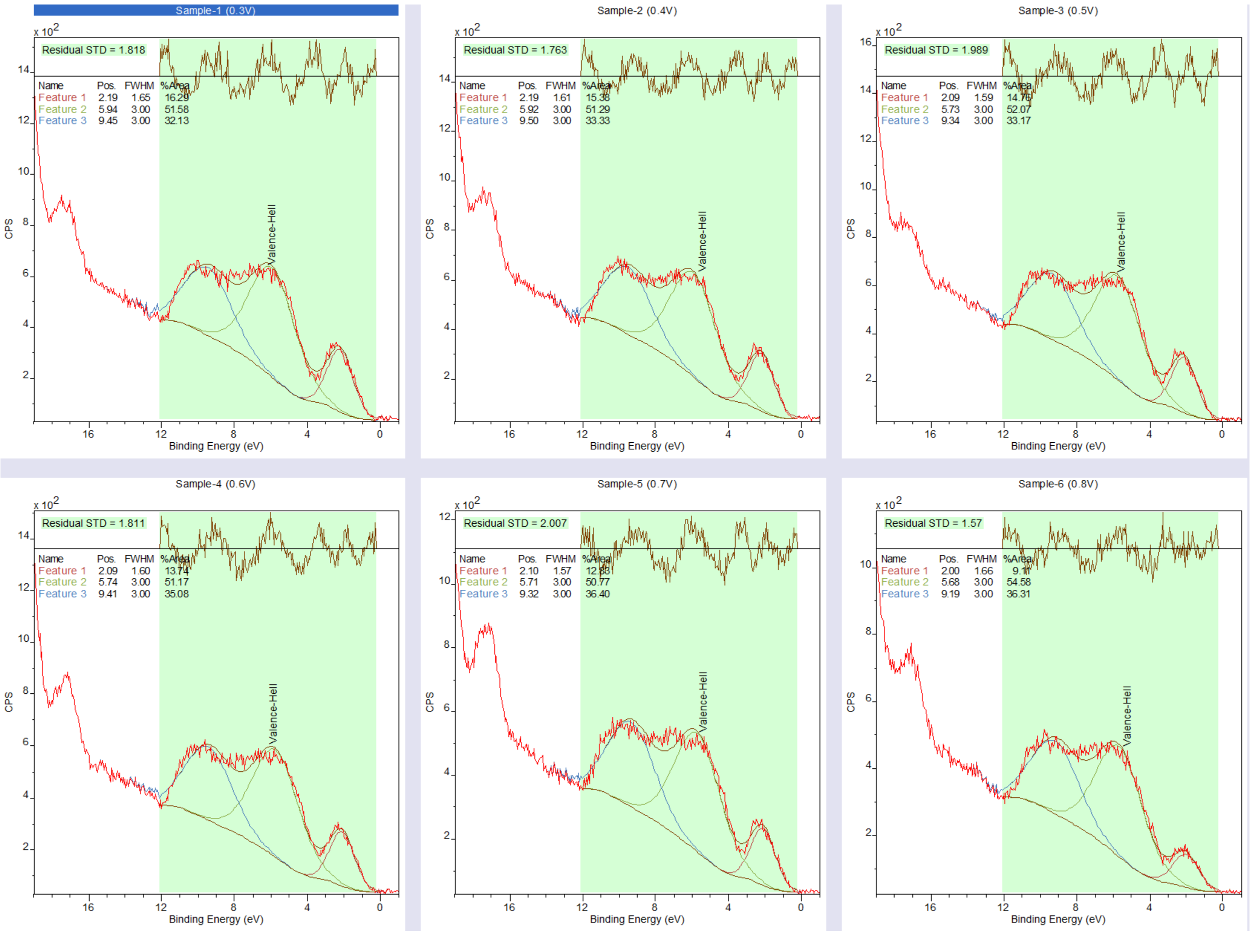

Figure S21. UPS HE II valence rough fits across the polarization range. Voltages can be found in title blocks.

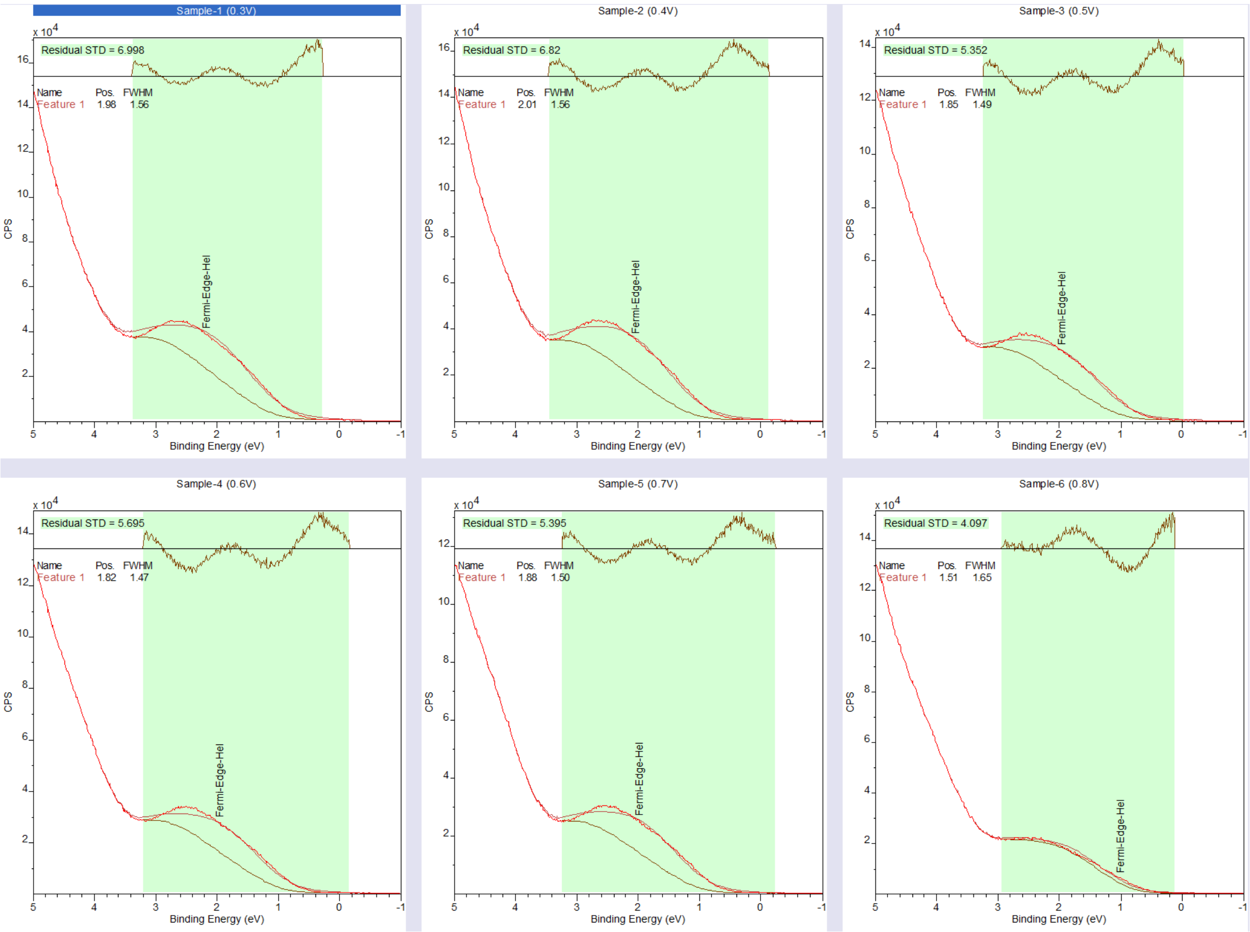

Figure S22. UPS HE II valence rough fits across the polarization range. Voltages can be found in title blocks.

References

1. H. A. Elsen, C. F. Monson, and M. Majda, "Effects of electrodeposition conditions and protocol on the properties of iridium oxide pH sensor electrodes," *Journal of The Electrochemical Society*, vol. 156, pp. F1-F6, 2009.
2. K. Yamanaka, "Anodically electrodeposited iridium oxide films (AEIROF) from alkaline solutions for electrochromic display devices," *Japanese journal of applied physics*, vol. 28, p. 632, 1989.
3. I. A. Lervik, M. Tsypkin, L.-E. Owe, and S. Sunde, "Electronic structure vs. electrocatalytic activity of iridium oxide," *Journal of Electroanalytical Chemistry*, vol. 645, pp. 135-142, 2010.
4. R. Kawar, P. Chigare, and P. Patil, "Substrate temperature dependent structural, optical and electrical properties of spray deposited iridium oxide thin films," *Applied Surface Science*, vol. 206, pp. 90-101, 2003.
5. G. Greczynski and L. Hultman, "Reliable determination of chemical state in x-ray photoelectron spectroscopy based on sample-work-function referencing to adventitious carbon: resolving the myth of apparent constant binding energy of the C 1s peak," *Applied Surface Science*, vol. 451, pp. 99-103, 2018.
6. G. Greczynski and L. Hultman, "C 1s peak of adventitious carbon aligns to the vacuum level: dire consequences for material's bonding assignment by photoelectron spectroscopy," *ChemPhysChem*, vol. 18, pp. 1507-1512, 2017.
7. M. Wojdyr, "Fityk: a general-purpose peak fitting program," *Journal of Applied Crystallography*, vol. 43, pp. 1126-1128, 2010.
8. K. Kanaya and S. Okayama, "Penetration and energy-loss theory of electrons in solid targets," *Journal of Physics D: Applied Physics*, vol. 5, p. 43, 1972.
9. J. Seiter, E. Müller, H. Blank, H. Gehrke, D. Marko, and D. Gerthsen, "Backscattered electron SEM imaging of cells and determination of the information depth," *Journal of microscopy*, vol. 254, pp. 75-83, 2014.
10. L. Burke and R. Scannell, "The effect of UV light on the hydrous oxides of iridium," *Journal of electroanalytical chemistry and interfacial electrochemistry*, vol. 257, pp. 101-121, 1988.
11. R. D. Smith, B. Sporinova, R. D. Fagan, S. Trudel, and C. P. Berlinguette, "Facile photochemical preparation of amorphous iridium oxide films for water oxidation catalysis," *Chemistry of Materials*, vol. 26, pp. 1654-1659, 2014.
12. D.-H. Kim, S.-H. Park, J. Choi, M. H. Yi, and H.-S. Kim, "Fabrication of iridium oxide nanoparticles supported on activated carbon powder by flashlight irradiation for oxygen evolutions," *Materials Science and Engineering: B*, vol. 201, pp. 29-34, 2015.
13. S. Ferro, D. Rosestolato, C. A. Martínez-Huitle, and A. De Battisti, "Charge-storage process in IrO_2-SnO_2 mixed-oxide electrodes. Role of coating composition, solution pH and Temperature," *Electrochimica Acta*, vol. 148, pp. 85-92, 2014.

14. Z. Pavlovic, C. Ranjan, Q. Gao, M. van Gastel, and R. Schlögl, "Probing the structure of a water-oxidizing anodic iridium oxide catalyst using Raman spectroscopy," *ACS Catalysis,* vol. 6, pp. 8098-8105, 2016.
15. C. Costentin, M. Robert, and J.-M. Savéant, "Electrochemical concerted proton and electron transfers. Potential-dependent rate constant, reorganization factors, proton tunneling and isotope effects," *Journal of Electroanalytical Chemistry,* vol. 588, pp. 197-206, 2006.
16. J.-C. Dupin, D. Gonbeau, P. Vinatier, and A. Levasseur, "Systematic XPS studies of metal oxides, hydroxides and peroxides," *Physical Chemistry Chemical Physics,* vol. 2, pp. 1319-1324, 2000.
17. E. McCafferty and J. Wightman, "Determination of the concentration of surface hydroxyl groups on metal oxide films by a quantitative XPS method," *Surface and Interface Analysis: An International Journal devoted to the development and application of techniques for the analysis of surfaces, interfaces and thin films,* vol. 26, pp. 549-564, 1998.
18. R. Kötz, H. Neff, and S. Stucki, "Anodic Iridium Oxide Films: XPS-Studies of Oxidation State Changes and," *Journal of the Electrochemical Society,* vol. 131, p. 72, 1984.
19. A. Cruz, L. Abad, N. Carretero, J. Moral-Vico, J. Fraxedas, P. Lozano, *et al.*, "Iridium oxohydroxide, a significant member in the family of iridium oxides. Stoichiometry, characterization, and implications in bioelectrodes," *The Journal of Physical Chemistry C,* vol. 116, pp. 5155-5168, 2012.
20. T. Yano, M. Ebizuka, S. Shibata, and M. Yamane, "Anomalous chemical shifts of Cu 2p and Cu LMM Auger spectra of silicate glasses," *Journal of electron spectroscopy and related phenomena,* vol. 131, pp. 133-144, 2003.
21. S. J. Freakley, J. Ruiz-Esquius, and D. J. Morgan, "The X-ray photoelectron spectra of Ir, IrO_2 and $IrCl_3$ revisited," *Surface and Interface Analysis,* vol. 49, pp. 794-799, 2017.
22. Y. Ping, R. J. Nielsen, and W. A. Goddard III, "The reaction mechanism with free energy barriers at constant potentials for the oxygen evolution reaction at the IrO_2 (110) surface," *Journal of the American Chemical Society,* vol. 139, pp. 149-155, 2017.
23. A. Zagalskaya and V. Alexandrov, "Mechanistic Study of IrO_2 Dissolution during the Electrocatalytic Oxygen Evolution Reaction," *The Journal of Physical Chemistry Letters,* vol. 11, pp. 2695-2700, 2020.
24. D. Hong, M. Murakami, Y. Yamada, and S. Fukuzumi, "Efficient water oxidation by cerium ammonium nitrate with [Ir III (Cp*)(4, 4′-bishydroxy-2, 2′-bipyridine)(H 2 O)] 2+ as a precatalyst," *Energy & Environmental Science,* vol. 5, pp. 5708-5716, 2012.
25. V. Pfeifer, T. E. Jones, J. J. Velasco Vélez, C. Massué, R. Arrigo, D. Teschner, *et al.*, "The electronic structure of iridium and its oxides," *Surface and Interface Analysis,* vol. 48, pp. 261-273, 2016.
26. V. Pfeifer, T. E. Jones, J. J. V. Vélez, R. Arrigo, S. Piccinin, M. Hävecker, *et al.*, "In situ observation of reactive oxygen species forming on oxygen-evolving iridium surfaces," *Chemical science,* vol. 8, pp. 2143-2149, 2017.
27. R. Sachse, M. Pflüger, J.-J. Velasco-Vélez, M. Sahre, J. Radnik, M. Bernicke, *et al.*, "Supporting Information Assessing optical and electrical properties of highly active IrOx catalysts for the electrochemical oxygen evolution reaction via spectroscopic ellipsometry."
28. L. Atanasoska, R. Atanasoski, and S. Trasatti, "XPS and AES study of mixed layers of RuO_2 and IrO_2," *Vacuum,* vol. 40, pp. 91-94, 1990.
29. J. Kahk, C. Poll, F. Oropeza, J. Ablett, D. Céolin, J. Rueff, *et al.*, "Understanding the Electronic Structure of IrO 2 Using Hard-X-ray Photoelectron Spectroscopy and Density-Functional Theory," *Physical review letters,* vol. 112, p. 117601, 2014.
30. A. Bourlange, D. Payne, R. Palgrave, H. Zhang, J. Foord, R. Egdell, *et al.*, "The influence of Sn doping on the growth of In 2 O 3 on Y-stabilized ZrO 2 (100) by oxygen plasma assisted molecular beam epitaxy," *Journal of Applied Physics,* vol. 106, p. 013703, 2009.
31. D. J. Payne, R. G. Egdell, W. Hao, J. S. Foord, A. Walsh, and G. W. Watson, "Why is lead dioxide metallic?," *Chemical Physics Letters,* vol. 411, pp. 181-185, 2005.
32. N. X. r. P. S. Database, "NIST Standard Reference Database Number 20, National Institute of Standards and Technology, Gaithersburg MD, 20899," 2000.
33. J. Hämäläinen, M. Kemell, F. Munnik, U. Kreissig, M. Ritala, and M. Leskelä, "Atomic layer deposition of iridium oxide thin films from Ir (acac) 3 and ozone," *Chemistry of Materials,* vol. 20, pp. 2903-2907, 2008.
34. Z.-M. Zhang, S. Chen, and Y.-Z. Liang, "Baseline correction using adaptive iteratively reweighted penalized least squares," *Analyst,* vol. 135, pp. 1138-1146, 2010.

Disclaimer/Publisher's Note: The statements, opinions and data contained in all publications are solely those of the individual author(s) and contributor(s) and not of MDPI and/or the editor(s). MDPI and/or the editor(s) disclaim responsibility for any injury to people or property resulting from any ideas, methods, instructions or products referred to in the content.
